# Tigecycline Opposes Bortezomib Effect on Myeloma Cells Decreasing Mitochondrial Reactive Oxygen Species Production

**DOI:** 10.3390/ijms25094887

**Published:** 2024-04-30

**Authors:** Carlos Ramos-Acosta, Laura Huerta-Pantoja, Milton Eduardo Salazar-Hidalgo, Elsa Mayol, Selene Jiménez-Vega, Pablo García-Peña, Jenifeer Jordi-Cruz, Cristina Baquero, Almudena Porras, Belén Íñigo-Rodríguez, Celina M. Benavente, Andrea R. López-Pastor, Irene Gómez-Delgado, Elena Urcelay, Francisco Javier Candel, Eduardo Anguita

**Affiliations:** 1 Department of Medicine, Medical School, Universidad Complutense de Madrid (UCM), Plaza Ramón y Cajal s/n, 28040 Madrid, Spainemayol@ucm.es (E.M.); selenejv9712@gmail.com (S.J.-V.); jenjordi@ucm.es (J.J.-C.); celinamaria.benavente@salud.madrid.org (C.M.B.); fj.candel@gmail.com (F.J.C.); 2 Hematology Department, IML, Instituto de Investigación Sanitaria San Carlos (IdISSC), Hospital Clínico San Carlos, Profesor Martín Lagos s/n, 28040 Madrid, Spainbelen.inigoro@salud.madrid.org (B.Í.-R.); 3 Department of Biochemistry and Molecular Biology, Faculty of Pharmacy, Universidad Complutense de Madrid (UCM), Instituto de Investigación Sanitaria San Carlos (IdISSC), 28040 Madrid, Spain; cbaque01@ucm.es (C.B.); maporras@ucm.es (A.P.); 4 Laboratory of Genetics and Molecular Bases of Complex Diseases, Instituto de Investigación Sanitaria San Carlos (IdISSC), 28040 Madrid, Spain; andrea.raposo@salud.madrid.org (A.R.L.-P.); igomezd@salud.madrid.org (I.G.-D.); elena.urcelay@salud.madrid.org (E.U.); 5 Networks for Cooperative Research in Health Results (RICORS, REI), 28089 Madrid, Spain; 6 Clinical Microbiology & Infectious Diseases, Transplant Coordination, IML, Instituto de Investigación Sanitaria San Carlos (IdISSC), Hospital Clínico San Carlos, 28040 Madrid, Spain

**Keywords:** multiple myeloma, tigecycline, bortezomib, ROS, cell cycle, antagonism

## Abstract

Multiple myeloma is an incurable plasma cell malignancy. Most patients end up relapsing and developing resistance to antineoplastic drugs, like bortezomib. Antibiotic tigecycline has activity against myeloma. This study analyzed tigecycline and bortezomib combination on cell lines and plasma cells from myeloma patients. Apoptosis, autophagic vesicles, mitochondrial mass, mitochondrial superoxide, cell cycle, and hydrogen peroxide were studied by flow cytometry. In addition, mitochondrial antioxidants and electron transport chain complexes were quantified by reverse transcription real-time PCR (RT-qPCR) or western blot. Cell metabolism and mitochondrial activity were characterized by Seahorse and RT-qPCR. We found that the addition of tigecycline to bortezomib reduces apoptosis in proportion to tigecycline concentration. Supporting this, the combination of both drugs counteracts bortezomib in vitro individual effects on the cell cycle, reduces autophagy and mitophagy markers, and reverts bortezomib-induced increase in mitochondrial superoxide. Changes in mitochondrial homeostasis and *MYC* upregulation may account for some of these findings. These data not only advise to avoid considering tigecycline and bortezomib combination for treating myeloma, but caution on the potential adverse impact of treating infections with this antibiotic in myeloma patients under bortezomib treatment.

## 1. Introduction

Multiple myeloma (MM) is a neoplasm of terminally differentiated plasma cells characterized by the production of a monoclonal immunoglobulin or immunoglobulin light chain (called M protein) [1]. According to the International Myeloma Working Group (IMWG), MM is defined by ≥10% clonal bone marrow plasma cells and one or more myeloma-defining events [2]. MM is almost always preceded by an asymptomatic premalignant disorder named monoclonal gammopathy of undetermined significance (MGUS) [1,2], and smoldering myeloma, an intermediate condition between MGUS and MM. Both disorders must lack myeloma-defining criteria [1,2] and are not usually treated. Other plasma cell neoplasms include solitary plasmacytoma, a condition with a single localized bone or extraosseous tumor formed by monoclonal plasma cells with <10% bone marrow clonal plasma cell infiltration and no myeloma-defining events [1].

MM is the third most frequent hematological malignancy, with a worldwide incidence of 176,404 in 2020. Furthermore, there is an increasing trend of MM incidence, predominantly in men, 50 years or older, and in high-income countries [3]. Thus, estimated new MM cases in the USA for 2024 are 35,780 [4]. Present treatments allow for an increase in the survival of patients suffering from MM. However, MM eventually relapses and today this is still considered an incurable disease [1]. Therefore, it is very important to develop more effective treatment strategies and to understand the mechanisms of resistance against them, which also requires a deep understanding of the pathogenesis of this condition.

Reactive oxygen species (ROS) have a key role in cancer, promoting survival and proliferation of tumor cells. Antioxidant enzymes are essential to avoid cell damage due to excessive ROS concentrations [5]. Hydrogen peroxide (H_2_O_2_) and superoxide anion (O_2_^•−^) are the most important ROS components in cancer. O_2_^•−^ is converted to H_2_O_2_ by superoxide dismutases (SODs) and then H_2_O_2_ is detoxified to H2O by catalase (CAT), glutathione peroxidase (GPX), and peroxiredoxin (PRDX) [5]. Antioxidant enzymes function is complex, as exemplified by the action of the major cytosolic superoxide dismutase SOD1 in the nucleus [6,7] and repressing respiration [8]. Unlike SOD1, SOD2 is localized in the mitochondrial matrix, protecting mitochondria against oxidative damage. Consistently, SOD2 is expressed at high levels in late-stage tumors. This increased expression of SOD2 can induce a metabolic switch from mitochondrial respiration to glycolysis in cancer cells [9]. Additionally, the regulator of the redox signaling pathways thioredoxin (TXN) system has emerged as an important regulator of cancer development [5]. Due to the malignant nature of their illness, MM patients have raised oxidative stress [10]. Also, increased expression of antioxidants including TXN, SOD1, and PRDX6 has been found in MM plasma cells compared to their normal counterparts. Moreover, patients with high levels of the last two show adverse outcomes [11,12]. Further highlighting the importance of ROS, NFE2-Like BZIP Transcription Factor 2 (NFE2L2), which is a key regulator of cellular antioxidant response, is inhibited in MM with t(4;14), increasing ROS and MM cell proliferation [13].

Mitochondria are essential in neoplastic development as they are the major producers of cellular oxidants and are involved in processes such as oxidative phosphorylation, the triggering of apoptosis, calcium homeostasis, and responses to oxidative stress [14]. The importance of mitochondria in the pathogenesis of monoclonal gammopathies is exemplified by the increase in mitochondrial DNA (mtDNA) copy number in MM cells compared with healthy individuals’ plasma cells and the upregulation of genes related to mitochondrial biogenesis in parallel with the disease progression [15].

Autophagy aids cells in getting rid of damaged organelles including mitochondria and misfolded proteins, which is particularly important in neoplastic plasma cells that produce huge amounts of immunoglobulin. Hence, MM cells have high levels of autophagy and are highly dependent of it for survival [16]. Paradoxically, some drugs can induce both autophagy and apoptosis in MM cells [17], and excessive activation of autophagy leads to autophagic cell death in MM [18].

Cell cycle abnormalities are also characteristically detected in cancer cells. In MM, cyclin D is overexpressed in most patients. This and other abnormalities of the cyclin D–CDK4/6–RB–INK4 pathway, frequently found in MM, promote G1/S transition. The dysregulation of these and other cell cycle components favor cell cycle progression and proliferation [19].

Proto-oncogene MYC is a master regulator of many of these functions, including transcription, apoptosis, ROS production, cell metabolism, and cell cycle progression [20,21]. Additionally, it has been involved in progression of MGUS to MM and MM prognosis [20].

Efficient treatment of MM is achieved with drug combinations; most of them include bortezomib (BTZ), a proteasome inhibitor that produces accumulation of misfolded proteins, eventually leading to apoptosis [22]. Redox homeostasis imbalance stands out as a major factor for both BTZ therapeutic activity and resistance [10,11,12]. Thus, ROS increase favors BTZ-induced cell death by apoptosis and autophagy, while ROS reduction antagonizes BTZ [12]. Also, the inhibition of SOD1 intensifies BTZ cytotoxicity and can reverse BTZ resistance. Conversely, SOD1 and GPX1 overexpression enhance BTZ resistance [9]. Mitochondria are also involved in the emergence of BTZ resistance [23,24,25,26,27,28]. Furthermore, BTZ increases autophagy, and its inhibition can antagonize BTZ cytotoxicity [16], suggesting that BTZ could lead to autophagic cell death [29]. Finally, BTZ has been shown to block cell cycle in G2/M phase, inducing apoptosis [22,30].

BTZ-intrinsic and acquired resistance [22] entails that new drugs are needed to increase BTZ therapeutic activity and avoid resistance. Thus, the impact of the antibiotic tigecycline (TIG), with anticancer activity through suppression of mitochondrial protein synthesis, and reduction in respiratory chain activity and oxygen consumption [31], has been recently analyzed in MM [32]. This study shows that TIG inhibits proliferation in MM cell lines, arresting cell cycle at G0/G1 [32]. Equally, the combination of BTZ and TIG has also been recently explored in MM cells in vitro and in animal models [33]. In contrast to the synergistic effect of this combination described with low TIG concentrations in the former study, we observed an antagonism in other cell models and primary human MM cells. The concern that BTZ and TIG combination might eventually be inefficient or even counterproductive in some patients urged us to further investigate the mechanism that may drive MM cell survival under this drug combination. This study underlines the importance of oxidative stress and cell metabolism in MM drug sensitivity.

## 2. Results

### 2.1. BTZ and TIG Individual Treatments Induce Apoptosis in KMS20 and KMS28BM Cells

We first aimed to test the differential effect of BTZ and TIG single treatment versus the combination of both drugs in BTZ-resistant KMS20 compared to BTZ-sensitive KMS28BM human MM cell lines [23].

**Figure 1:**
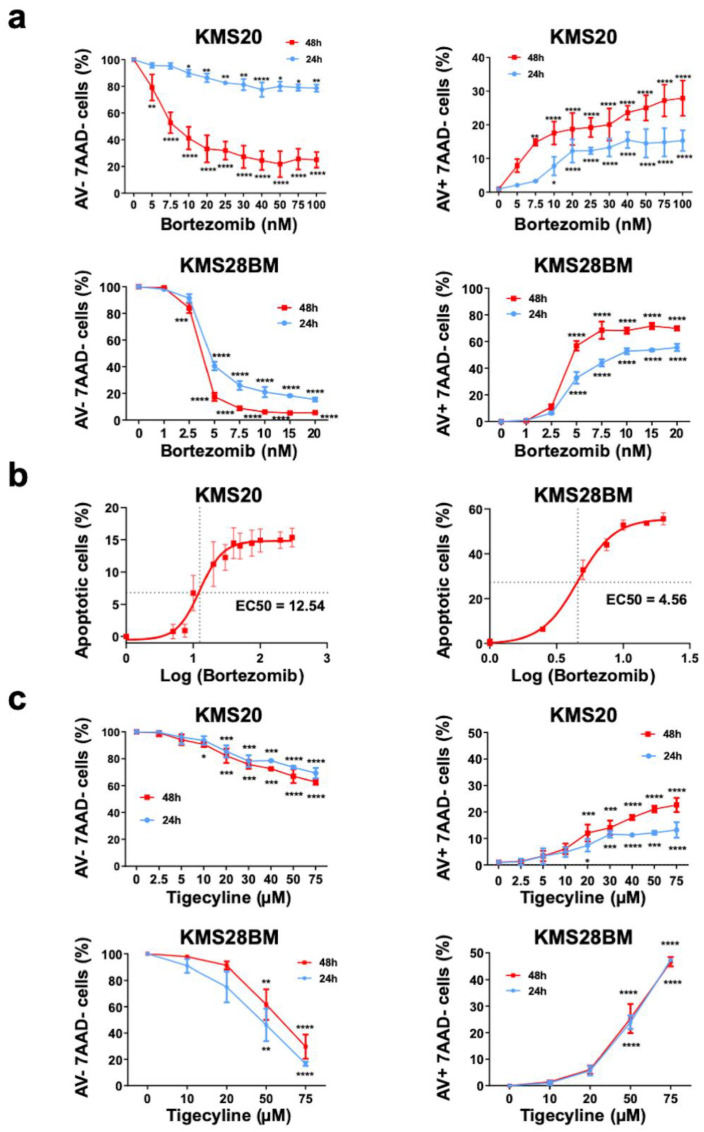
BTZ and TIG individually decrease cell viability in KMS20 and KMS28BM cells in a dose- and time-dependent manner. (**a**) Graphical depiction of cell viability and early apoptosis analysis of KMS20 (**top** panels) and KMS28BM cells (**bottom** panels) treated with increasing concentrations of BTZ, as indicated, for 24 h (in blue) or 48 h (in red) and analyzed by flow cytometry. On the left panels, cell viability represented by annexin V (AV) and 7AAD double negative cells. On the right, early apoptosis, AV positive/7AAD negative cells. Three biologically independent experiments were performed for each assay (2 technical replicas of each), and they were analyzed by one-way ANOVA. In the viability study, data of control cells incubated with vehicle (0) were set to 100 and in the apoptosis analysis control were fixed to 0; the results of the other culture conditions are expressed in relation to control cells. Plots show means ± SEM. * *p* < 0.05, ** *p* < 0.01, *** *p* < 0.001, and **** *p* < 0.0001. (**b**) Calculation of the half maximal effective concentration (EC50) of bortezomib in KMS20 (left) and KM28BM (right) cell lines. Log: logarithm. EC50 for each cell line is indicated. (**c**) Cell viability and early apoptosis analysis of KMS20 (**top** panels) and KMS28BM cells (**bottom** panels) treated with increasing concentrations of TIG, as indicated, for 24 h (in blue) or 48 h (in red) and analyzed by flow cytometry (n = 3–2). Plots and analysis as in (**a**).

It is known that BTZ reduces cellular viability and promotes apoptosis of MM plasma cells [22]. We confirmed these effects under our experimental conditions treating KMS20 and KMS28BM cells with increasing doses of BTZ (5–100 nM and 1–20 nM, respectively) for 24 and 48 h. As expected, we observed more potent effects on KMS28BM than KMS20 (Figure 1a). We determined the EC50 of both cell lines, 4.56 nM and 12.54 nM, respectively (Figure 1b). This reflected the expected resistance of KMS20 compared to KMS28BM.

TIG’s antimyeloma effect [32], together with its effect on mitochondria [31], prompted us to test the combination of this drug with BTZ in the context of KMS20 and KMS28BM cells, expecting to overcome BTZ resistance. To properly interpret this study, we first analyzed the proapoptotic effect of TIG as single treatment. It has been described that TIG induces apoptosis in some cellular models [34,35,36,37,38]. Conversely, it produced little apoptosis in other studies [17,25,32]. When we measured the percentage of apoptotic KMS20 cells after 24 and 48 h of exposure with increasing concentrations of TIG (0–75 µM), following published ranges [32,35,36,37,38,39], we observed that it rose as TIG concentration augmented. This effect was more patent with higher concentrations, and statistical significance was observed from 20 µM to the maximum concentration tested, 75 µM, after exposure for both 24 and 48 h in KMS20 cells. Similar data were obtained in KMS28BM cells with statistical significance from 50 µM TIG (Figure 1c).

### 2.2. TIG and BTZ Treatment Combination Shows Antagonistic Effects Reducing Cell Death

To study the effect of both drugs together, we used the EC50 of BTZ and rising concentrations of TIG.

**Figure 2:**
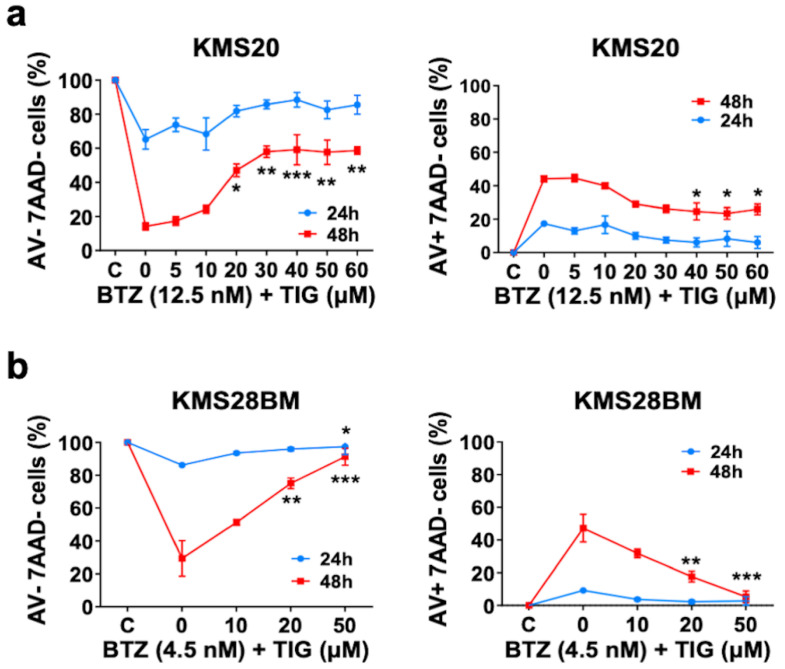
TIG and BTZ combination and BTZ treatment show opposed effects on cell death. (**a**) Cell viability and early apoptosis analysis of KMS20 cells studied by flow cytometry. On the left-hand side, plot of viable cells defined as annexin V (AV)/7AAD double-negative; control, C, is set to 100 and the results of the other culture conditions are expressed as a percentage of it (Y axis). On the right side, plot of early apoptotic cells displayed as annexin V-positive/7AAD-negative; results of the different conditions were compared to the control cells established as 0 (Y axis). In each experiment, cells were treated with their respective EC50 of BTZ only (0) or together with increasing TIG concentrations, as indicated on the X-axis. Control, C: vehicle (DMSO)-treated cells. Data for 24 h (in blue) and 48 h (in red) drug exposure are shown. Three biologically independent experiments were performed for each assay (2 technical replicas of each), and they were analyzed by one-way ANOVA comparing each drug concentration with BTZ single treatment data. Plots show means ± SEM. * *p* < 0.05, ** *p* < 0.01, and *** *p* < 0.001 (comparing BTZ single treatment with each BTZ plus TIG combination). (**b**) Plots of KMS28BM cell viability (left-hand side) and early apoptosis (right side) studies displayed and analyzed as in (**a**).

When we combined BTZ and TIG for 48 h, we observed an increase in viable cells (expressed as annexin V and 7 ADD double-negative cells) in proportion to TIG dose, compared to cells treated with BTZ EC50 only, reaching statistical significance in both KMS20 and KMS28BM cells at a TIG concentration of 20 µM (*p* = 0.0188 and *p* = 0.0013, respectively) or higher (Figure 2 and Figure S1). Likewise, the proportion of early apoptotic cells (annexin V-positive and 7 ADD-negative cells) significantly decreased in KMS20 from 40 µM (*p* = 0.0439) and in KMS28BM cells from 20 µM TIG (*p* = 0.0064) (Figure 2 and Figure S1). The effect after 24 h had a similar tendency without reaching statistical significance, except for annexin V and 7 ADD double-negative KMS28BM cells treated with BTZ EC50 and 50 µM TIG (*p* = 0.0404) (Figure 2b).

**Figure 3:**
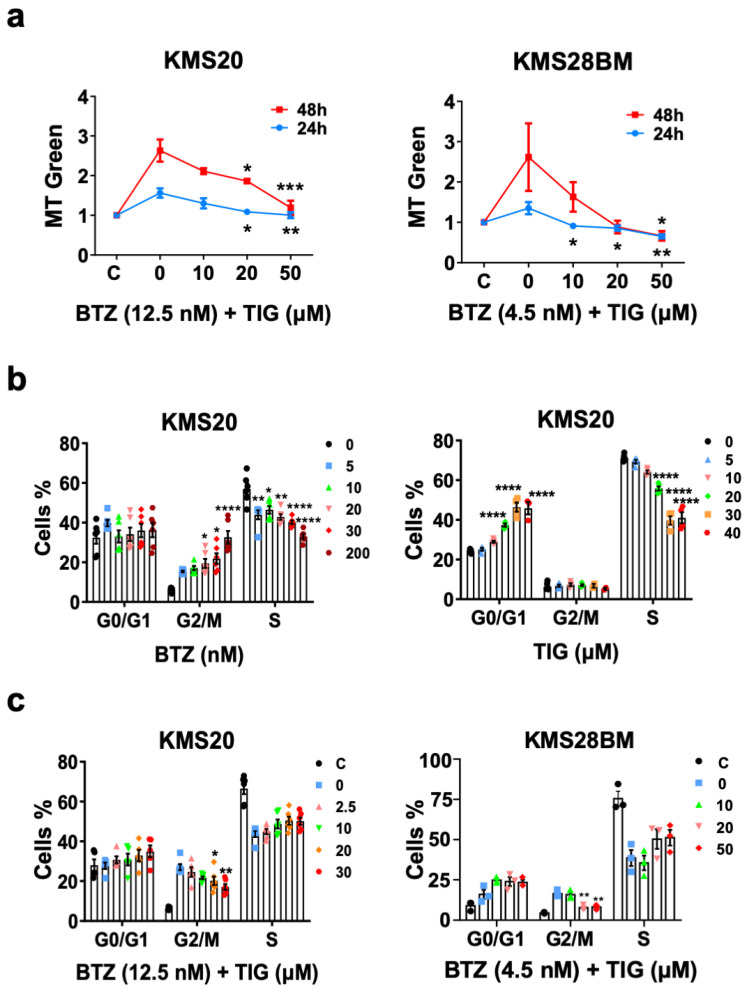
Apoptosis reduction produced by TIG plus BTZ combination versus BTZ single treatment correlates with mitochondrial mass decrease and associates to cell cycle changes. (**a**) Mitochondrial mass, assessed with MitoTracker Green staining and flow cytometry analysis. In each experiment, cells were treated with vehicle (DMSO), their respective EC50 of BTZ only (0), or BTZ EC50 together with increasing TIG concentrations, as indicated. On the left-hand side, plot of the analysis on KMS20 cells. Data of control, DMSO-treated cells (C) were fixed to 1 and the results of the different conditions referred to it. Data for 24 h (in blue) and 48 h (in red) drug exposure are shown. Three biologically independent experiments were performed for each assay (2 technical replicas of each), and they were analyzed by one-way ANOVA comparing each drug concentration with BTZ single treatment data. Plot shows means ± SEM. * *p* < 0.05, ** *p* < 0.01, and *** *p* < 0.001. On the right-hand side, plot of the study on KMS28BM cells performed and analyzed as before. (**b**) Cell cycle study of KMS20 cells by flow cytometry using APC BrdU Flow kit after 48 h of single drug treatment. Plots of data obtained from cells incubated with increasing concentrations of BTZ (left-hand side panel) and tigecycline (right-hand side panel) are shown. Control, C: vehicle (DMSO)-treated cells (black dots). Statistical analysis as before. Error bars indicate means ± SEM of three independent experiments. * *p* < 0.05, ** *p* < 0.01, and **** *p* < 0.0001. (**c**) Cell cycle study of BTZ and TIG combined treatment of KMS20 (left-hand side panel) and KMS28BM (right-hand side panel) cells studied by flow cytometry, analyzed and plotted as in (**b**). Error bars indicate means ± SEM of three independent experiments. * *p* < 0.05 and ** *p* < 0.01.

Higher mitochondrial level has been associated to increased susceptibility to apoptosis in cancer cells [40]. Concordantly, we observed an increase in mitochondrial mass after BTZ treatment. This effect was reverted by the addition of TIG for both 24 and 48 h in KMS20 and KMS28BM, reaching significance at concentrations of 10–20 µM (Figure 3a and Figure S2a).

The proapoptotic effect of BTZ has been related to G2/M-phase cell cycle arrest [30]. Accordingly, the percentage of KMS20 cells at G2/M phase increased and cells at S phase reduced in proportion to the concentration of BTZ (Figure 3b and Figure S2b). In contrast, TIG has been shown to arrest the cell cycle at G0/G1 in MM cells [32], among other malignant cells [37,41], although arrest at G2/M has also been described [37,42]. We found that TIG increased levels of KMS20 cells at G0/G1 phase and decreased cells in S phase, reaching significance at 20 µM and a plateau at 30 µM in both cell cycle phases (Figure 3b). Thus, we wondered if the different impact on the cell cycle could produce an interference between both drugs. In fact, the addition of TIG produced a reduction of cells at G2/M phase compared to BTZ individual treatment, reaching significance with a 20 µM concentration in KMS20 and KMS28BM (*p* = 0.0395 and *p* = 0.0013, respectively) and a tendency to increase cells at S phase in both cell lines (Figure 3b and Figure S2b).

In summary, we observed that the addition of TIG to BTZ can reduce apoptosis and partly counteract the effect of BTZ at the cell cycle, rendering this association antagonistic in the studied MM cells.

### 2.3. TIG Neutralizes BTZ Driven Increased Autophagy

**Figure 4:**
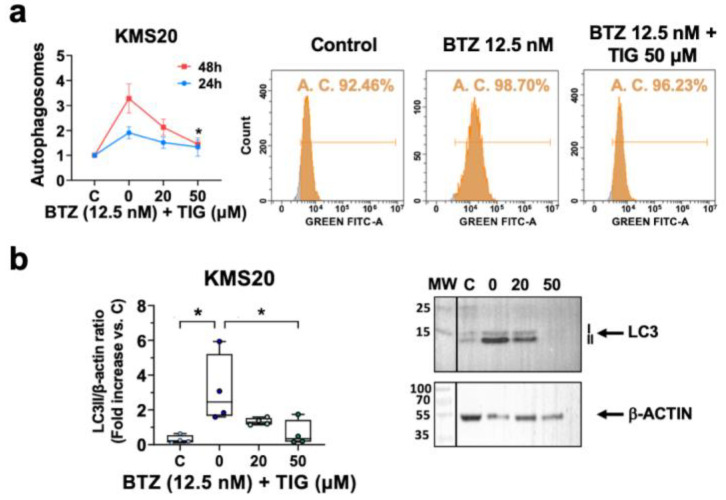

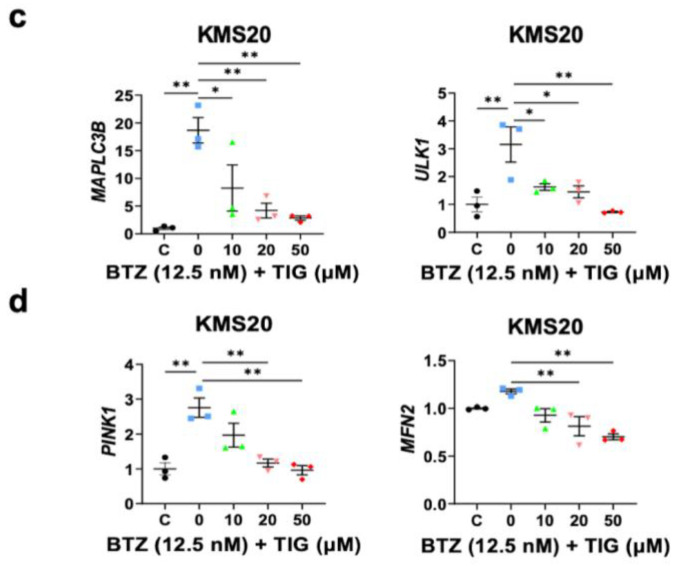
The addition of TIG to BTZ reverses BTZ-linked increased autophagy markers. (**a**) Left, plot of the mean fluorescence signal of monodansylcadaverine (MDC)-stained autophagic vacuoles (autophagosomes) assessed by flow cytometry of KMS20 cells treated with four different conditions: C, DMSO control; 0, BTZ EC50 (12.5 nM); 20, 12.5 nM BTZ plus 20 μM TIG; and 50, 12.5 nM BTZ with 50 μM TIG. Means ± SEM of 3 independent experiments after 24 h and 48 h drug incubation are shown in blue and red, respectively, * *p* < 0.05 (comparing BTZ single treatment with each BTZ plus TIG combination). Right, representative flow cytometry study, for simplicity only control (DMSO), BTZ EC50 (12.5 nM), and 12.5 nM BTZ plus 50 μM TIG after 48 h incubation are shown. A. C., autophagic cells (**b**) Levels of autophagy central protein LC3B analyzed by western blot. Left hand side, quantification of LC3B-II normalized with beta actin in KMS20 cells, after 48 h drug incubation as in (**a**) (n = 3). Western blot densitometry analysis was performed using ImageJ Fiji win64 software. Results are expressed using box and whiskers diagrams, where lines represent the median value, boxes the 25th and 75th percentiles, and whiskers mark maximum and minimum values, * *p* < 0.05. Right hand side, representative western blot showing LC3B (forms I and II, as indicated) and beta actin (β-ACTIN) control. MW: molecular weight marker from the same gel electrophoresis cut from its original loading position; C: DMSO (vehicle) control. (**c**) RNA expression of autophagy key genes *MAP1LC3B*, coding for LC3B, (left) and *ULK1* (right) quantified by RT-qPCR in KMS20 cells after 48 h drug exposure as in (**a**). Levels were normalized with both *TBP* and *RPL30* expression with similar results; for simplicity, only *TBP* normalized data are shown. Error bars indicate means ± SEM of three independent experiments, * *p* < 0.05, and ** *p* < 0.01. (**d**) RNA expression of mitophagy master genes *PINK1* (left) and *MFN2* (right). Analysis and plots as in (**c**).

Autophagy has a controversial role in MM. Thus, it may be an important mechanism driving malignant plasma cell survival and BTZ resistance, but can also mediate cell death [10,29]. To gain insight on the role of autophagy in previously observed phenomena, we analyzed the formation of autophagic vacuoles by flow cytometry. These significantly increased with BTZ treatment as a single drug compared to vehicle (DMSO) control (*p* = 0.0069), but reduced with the addition of TIG, reaching significance with 50 µM concentration exposure for 48 h compared to BTZ EC50 only (*p* = 0.0224) (Figure 4a). Autophagy inhibition in the combined treatment was confirmed by western blot analysis of protein levels of the autophagosome marker MAP1LC3B-II (LC3B-II), which followed the same pattern, being statistically significant (control vs. BTZ: *p* = 0.014 and BTZ vs. BTZ + TIG 50 µM: *p* = 0.031) (Figure 4b). Consistently, TIG significantly reduced the BTZ induced elevation of *MAP1LC3B* mRNA, and the key initiator of autophagy *ULK1* in both cell lines (Figure 4c and Figure S3a). We also explored if mitophagy in particular was affected in the same way. The expression of pivotal genes inducing mitophagy, *PINK1* and *MFN2* [43], also decreased significantly with the combined treatment in KMS20 (Figure 4d), showing the same tendency in KMS28BM (Figure S3b), which supports the idea that mitophagy diminishes with TIG association compared to BTZ alone.

In brief, these data suggest that the increase in apoptosis produced by the addition of TIG to BTZ is associated with a reduction in BTZ-driven autophagy and mitophagy.

### 2.4. TIG Reverses BTZ Dependent ROS Increase

Drug-mediated ROS increase produces MM cell death and is recognized as a mechanism to counteract proteasome inhibitors resistance [10]. Thus, we investigated the role of redox status in TIG-linked survival enhancement of BTZ-treated cells.

The major sources of ROS in a cell include mitochondria, endoplasmic reticulum, and peroxisomes [10].

**Figure 5:**
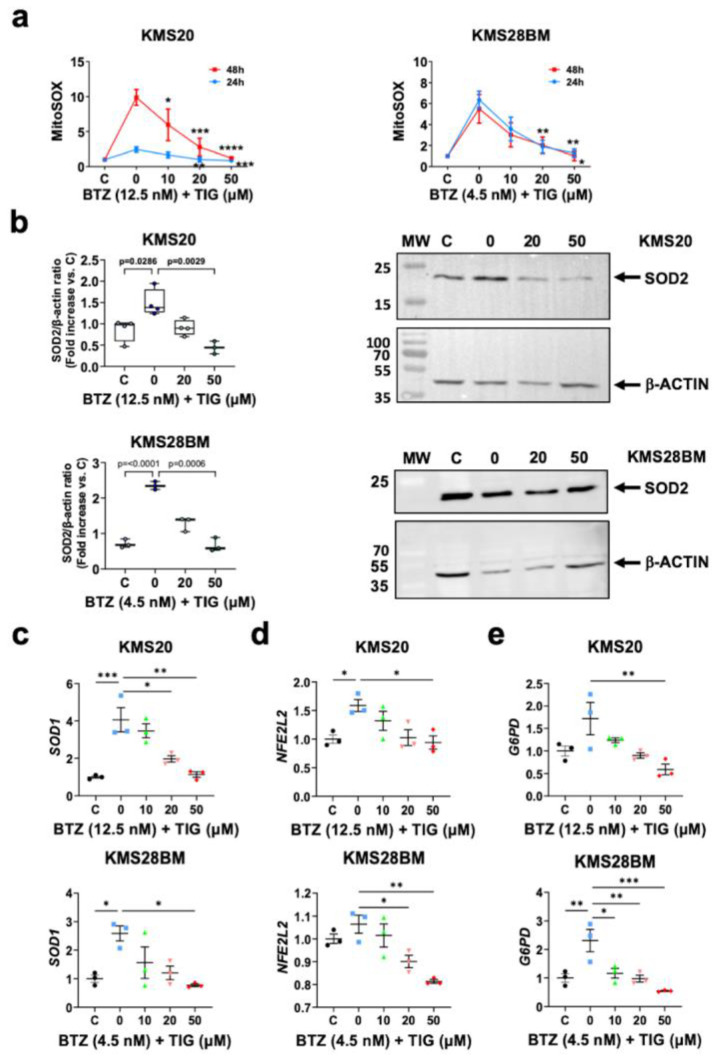
TIG reverses BTZ-dependent mitochondrial ROS increase without increased expression of superoxide antioxidants. (**a**) Analysis of superoxide production in mitochondria by MitoSOX Red flow cytometry assessment in KMS20 (left) and KMS28BM (right) cell lines. Cells were incubated with their EC50 of BTZ only (0) or with it plus increasing concentrations of TIG, as shown. C, control: vehicle-treated cells. Plots show means ± SEM of the data for 24 h (in blue) and 48 h (in red) culture in each condition. N = 3. * *p* < 0.05, ** *p* < 0.01, *** *p* < 0.001 and **** *p* < 0.0001. (**b**) SOD2 protein levels analyzed by western blot in KMS20 (**top**) and KMS28BM (**bottom**) cells after 48 h drug incubation as in (**a**). On the left panels, quantification of SOD2 levels normalized with beta actin, analysis using ImageJ Fiji win64 software (n = 3). Results are expressed using box and whiskers diagrams, where lines represent the median value, boxes the 25th and 75th percentiles, and whiskers mark maximum and minimum values; *p* values are shown. On the right, representative western blots for each cell line (KMS20 on the **top** and KMS28BM on the **bottom**) showing SOD2 and beta actin (β-ACTIN) control, as indicated. MW: molecular weight marker. C, control: vehicle-treated cells; 0, BTZ EC50 (12.5 nM); 20, 12.5 nM BTZ plus 20 μM TIG, and 50, 12.5 nM BTZ with 50 μM TIG. (**c**–**e**) mRNA expression of genes encoding antioxidant proteins *SOD1*, *NFE2L2,* and *G6PD*, respectively, analyzed by RT-qPCR as in Figure 4c. KMS20 cells are shown in the top panels and studies on KMS28BM in the bottom ones. Culture conditions for 48 h drug exposure and statistical significance as in (**a**). Plots show means ± SEM of 3 independent experiments.

First, we measured mitochondrial superoxide and observed augmented levels in both cell lines when we treated them with BTZ, KMS20 showing higher levels. TIG and BTZ combination produced a significant dose-dependent superoxide decrease in both cell lines (Figure 5a and Figure S4).

To obtain insight into the potential role of antioxidants in mitochondrial superoxide reduction and eventually the increase in cell survival with the combined treatment, we first analyzed mitochondrial SOD2. Although *SOD2* mRNA levels did not significantly change (Figure S5a), SOD2 protein levels significantly increased after BTZ single treatment in both cell lines (*p* = 0.0286 and *p* < 0.0001, in KMS20 and KMS28BM, respectively) and showed a dose-dependent reduction when TIG was added, reaching statistical significance with 50 µM TIG (*p* = 0.0029 and *p* = 0.0006, in KMS20 and KMS28BM, respectively) (Figure 5b). In agreement with this, cytoplasmic *SOD1* expression mirrored SOD2 expression behavior, compensating the increase produced by BTZ treatment in both cell lines (control vs. BTZ: *p* = 0.0007 and *p* = 0.0246 in KMS20 and KMS28BM, respectively, and BTZ vs. BTZ + TIG 50 µM: *p* = 0.001 and *p* = 0.0107, respectively). (Figure 5c). The expression of the *NFE2L2* gene, coding the master regulator of the cellular antioxidant response, including *SOD* expression [13,44], paralleled *SOD1* behavior. Additionally, the expression of cytoplasmic antioxidant *G6PD*, which is relevant in some MM models [45], showed overlapping results in both cell lines as before (Figure 5d).

In conclusion, TIG association to BTZ counteracts BTZ-induced mitochondrial ROS elevation.

### 2.5. The Combination of High TIG Concentrations to BTZ Increases Hydrogen Peroxide Levels

SOD enzymes catalyze the conversion of superoxide into H_2_O_2_ and O_2_. Thus, the finding of the reduction in mitochondrial superoxide and SOD levels with BTZ and TIG combination led us to study H_2_O_2_, which is also a major source of MM ROS at the endoplasmic reticulum in the process of immunoglobulin synthesis [10].

**Figure 6:**
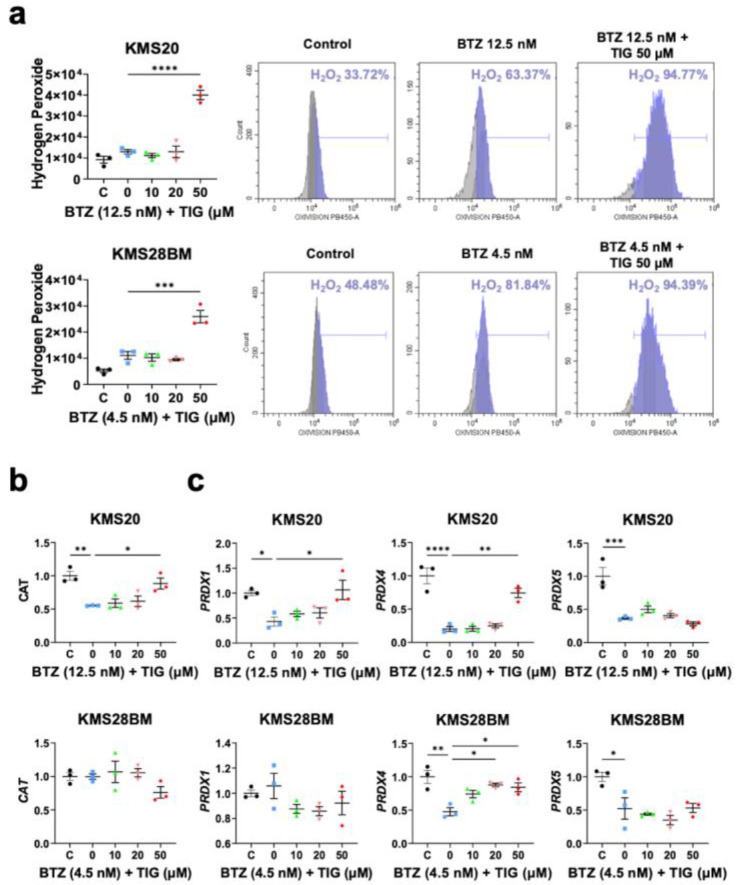
TIG plus BTZ treatment increases hydrogen peroxide and endoplasmic reticulum peroxiredoxin (*PRDX4*) expression in KMS20 and KMS28BM cells, while the expression of other H_2_O_2_ scavengers is variable, depending on the cell line, or decreases. (**a**) Hydrogen peroxide analysis by flow cytometry. On the left-hand side, plot representing the mean ± SEM of three experiments (two replicas of each) in KMS20 (**top**) and KMS28BM (**bottom**) after 24 h incubation with vehicle (control, C), BTZ EC50 (0) or BTZ EC50 plus the indicated concentration of TIG. *** *p* < 0.001 and **** *p* < 0.0001. On the right-hand side, representative flow cytometry analysis for each cell line. (**b**,**c**) mRNA expression of genes encoding H_2_O_2_ neutralizers analyzed by RT-qPCR as in Figure 4c. Culture conditions for 48 h drug exposure. Plots show means ± SEM of 3 independent experiments. * *p* < 0.05, ** *p* < 0.01, *** *p* < 0.001, and **** *p* < 0.0001. (**b**) Plots corresponding to *CAT* analysis; results of KMS20 cells are shown in the top panels and studies on KMS28BM in the bottom ones. (**c**) Peroxiredoxins studies (*PRDX1*, *PRDX4*, and *PRDX5*). Plots of data of KMS20 and KMS28BM cells are shown in the top and bottom panels, respectively.

H_2_O_2_ level sharply raised in both KMS20 and KMS28BM when 50 µM TIG was combined with BTZ EC50 (*p* < 0.0001 and *p* = 0.0002, respectively, compared to BTZ single treatment) with no increase using lower TIG concentrations (Figure 6a). Again, H_2_O_2_ levels were higher in KMS20 cells.

Next, we analyzed expression of the main genes encoding H_2_O_2_ scavengers acting at different cell compartments. CAT is mainly present at peroxisomes. In KMS20 cells, *CAT* mRNA levels significantly decreased in BTZ-treated cells compared to vehicle-treated control (*p* = 0.0047), and compared to BTZ single treatment, *CAT* raised after culturing these cells with BTZ and TIG 50 µM combination (*p* = 0.032). However, no significant changes in *CAT* levels were observed in KMS28BM cells (Figure 6b).

PRDXs are present in all subcellular compartments, but the different isoforms are predominantly present in specific locations [10]. Gene expression of the main cytosolic isoform *PRDX1* followed the *CAT* pattern (Figure 6b). In KMS20 *PRDX1* significantly decreased upon BTZ incubation compared to control (*p* = 0.0293) and significantly recovered with the addition of TIG (BTZ vs. BTZ + TIG 50 µM: *p* = 0.0157), but did not change in KMS28BM cells (Figure 6c). The expression of the isoform localized at the endoplasmic reticulum, *PRDX4,* significantly decreased upon BTZ treatment and significantly increased with the combination of BTZ and TIG to a level close to the control in both cell lines (control vs. BTZ: *p* < 0.0001 and *p* = 0.0017 in KMS20 and KMS28BM, respectively and BTZ vs. BTZ + TIG 50 µM: *p* = 0.0015 and *p* = 0.019, respectively). Finally, the expression of *PRDX5*, which encodes the isoenzyme present at the cytosol, mitochondria, and nucleus, was significantly reduced following BTZ treatment independently of the presence of TIG in the culture (control vs. BTZ: *p* = 0.0004 and *p* = 0.0252 in KMS20 and KMS28BM, respectively) (Figure 6c).

The thioredoxin system is necessary for the reduction of peroxiredoxins. Cytoplasmic and nuclear *TXN* mRNA levels significantly decreased after BTZ incubation compared to control (*p* = 0.006) and recovered with the combined treatment in KMS20 cells (BTZ vs. BTZ + TIG 50 µM: *p* = 0.0004). However, KMS28BM cells showed the opposite pattern: BTZ increased the expression (control vs. BTZ: *p* = 0.0108) and the combined treatment reduced *TXN* expression (BTZ vs. BTZ + TIG 50 µM: *p* = 0.0023). Instead, mitochondrial *TXN2* levels did not significantly change in the combined treatment compared to BTZ single treatment (Figure S5b). Transcript levels of *GPX1*, coding for a H_2_O_2_ scavenger present in both cytosolic and mitochondrial cell compartments did not change either (Figure S5b).

In summary, TIG association to BTZ increases H_2_O_2_, and the expression of hydrogen peroxide detoxifiers varies according to different cell lines and/or the compartment in which they are expressed.

### 2.6. BTZ-Linked Effects in Mitochondrial Function Are Reversed When TIG Is Associated to BTZ

Next, we explored if the reduction in mitochondrial superoxide production was associated to an improved mitochondrial homeostasis.

**Figure 7:**
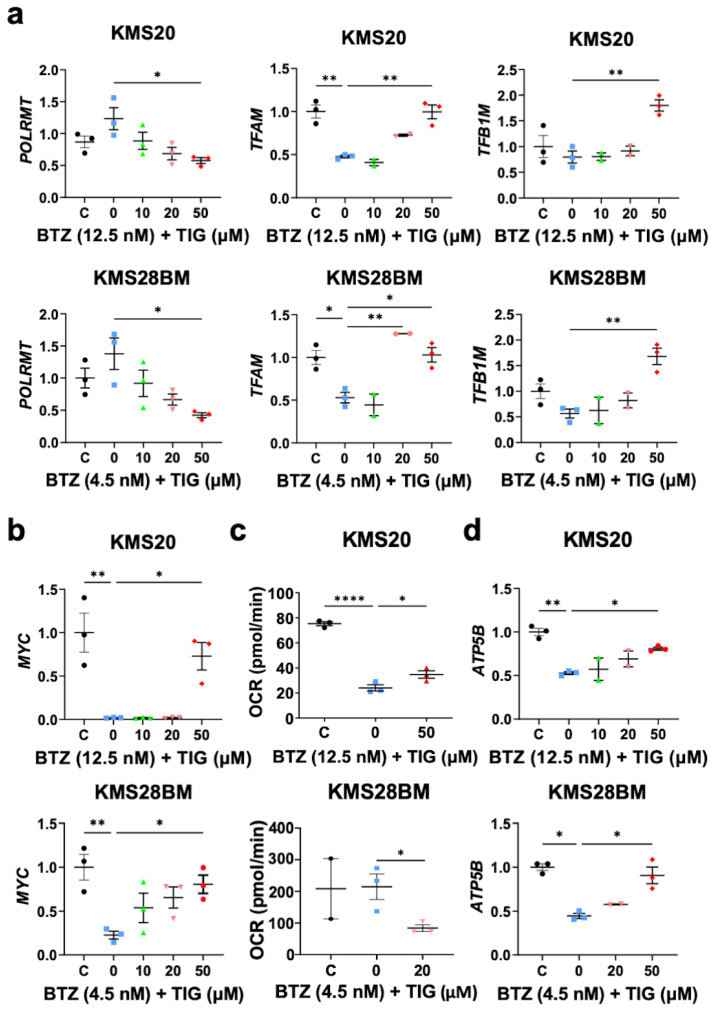
TIG counteracts BTZ effect on gene expression of key regulators of mitochondrial function. (**a**) *POLRMT*, *TFAM*, and *TFB1M* mRNA expression, as indicated, quantified by RT-qPCR in KMS20 (**top**) and KMS28BM (**bottom**) cells after 48 h incubation with either DMSO vehicle (C), the EC50 of BTZ for each cell line (0), or BTZ EC50 plus increasing concentrations of TIG, as shown. Plot and analysis as in Figure 4c, *TBP* normalized data are shown, error bars indicate means ± SEM of 2–3 independent experiments, * *p* < 0.05, ** *p* < 0.01. (**b**) *MYC* expression of in KMS20 (**top**) and KMS28BM (**bottom**) analyzed and shown as in (**a**) (n = 3). (**c**) Basal oxygen consumption rate (OCR) study in KMS20 (**top**) and KMS28BM (**bottom**) cells incubated for 48 h with either vehicle (C); the EC50 of BTZ or EC50 of BTZ plus tigecycline at the concentration that induce significant apoptosis in combination with BTZ, as indicated. Plots show means ± SEM basal respiration (before addition of oligomycin) of 2–3 independent experiments, * *p* < 0.05 and **** *p* < 0.0001. (**d**) *ATP5B* mRNA quantification in KMS20 (**top**) and KMS28BM (**bottom**). Culture conditions, graphs, and analysis as in (**a**).

First, we studied the expression of mitochondrial biogenesis and metabolism related genes. Thus, we analyzed the mRNA levels of the central gene for mitochondrial gene expression and mtDNA replication *POLRMT* (RNA Polymerase Mitochondrial). Following mitochondrial mass data, TIG reduced *POLRMT* expression in the in BTZ-treated KMS20 and KMS28BM cells (BTZ vs. BTZ + TIG 50 µM: *p* = 0.0171 and *p* = 0.0219 and in KMS20 and KMS28BM, respectively) (Figure 7a). Initiation of transcription by POLRMT is aided by transcription factor A, mitochondrial (TFAM) [46]. BTZ significantly reduced *TFAM* expression and the co-culture with TIG recovered it to control levels (control vs. BTZ: *p* = 0.0013 and *p* = 0.0398 in KMS20 and KMS28BM, respectively, and BTZ vs. BTZ + TIG 50 µM: *p* = 0.0014 and *p* = 0.0273 and in KMS20 and KMS28BM, respectively). Similarly, the mRNA levels of transcription factor B1 mitochondrial (TFB1M), mainly involved in the regulation of mitochondrial gene translation [47], increased with BTZ plus TIG combination (BTZ vs. BTZ + TIG 50 µM: *p* = 0.0055 and *p* = 0.0039 and in KMS20 and KMS28BM, respectively) (Figure 7a). All these data suggest that the drug combination affects mitochondrial function.

*MYC* proto-oncogene has multiple roles, including the promotion of mitochondrial function, cell cycle progression, and apoptosis [20,21]. It has been previously shown that BTZ reduces MYC expression in JJN3 MM cell line. When these cells were cultured with low doses of TIG (3.7 µM) and BTZ, MYC further decreased compared to BTZ single treatment [33]. We confirmed *MYC* mRNA reduction under BTZ treatment (control vs. BTZ: *p* = 0.0016 and *p* = 0.0089 in KMS20 and KMS28BM, respectively) (Figure 7b). Interestingly, in KMS20 cells *MYC* remained at very low levels with BTZ EC50 and 10–20 µM TIG combined treatment, but showed a significant rampant increase with 50 µM TIG dose (*p* = 0.0148). In contrast, KMS28BM cells exhibited a tendency to progressively increase *MYC* levels in parallel to TIG dose, reaching statistical significance with 50 µM (*p* = 0.0488) (Figure 7b). This suggests that the impact of TIG on *MYC* expression can be both dose- and cell-dependent. The expression of other important genes for mitochondrial homeostasis, *MTOR*, *AKT1*, and *OPA1*, did not significantly change (Figure S6).

Then, we studied mitochondrial respiration under BTZ single treatment and the combination of BTZ and TIG. The results appear to be cell type-dependent. In KMS20 the analysis of the OCR showed that BTZ reduced mitochondria basal respiration (*p* < 0.0001) and TIG partly counteracted this effect (*p* = 0.0493). However, in KMS28BM, the combination reduced the basal respiration compared to BTZ single treatment (*p* = 0.036) (Figure 7c). We also studied the basal ECAR bioenergetic profile as a measurement of glycolysis. In KMS20, BTZ reduced ECAR (*p* = 0.0129), but the addition of TIG did not significantly change it (Figure S7). No statistically significant differences were found in KMS28BM (Figure S7). To better clarify the impact of the combined treatment on the function of the mitochondria, we studied the expression of ATP Synthase F1 Subunit Beta (*ATP5B*). *ATP5B* transcripts dropped when cells were cultured with BTZ (control vs. BTZ: *p* = 0.00709 and *p* = 0.0172 in KMS20 and KMS28BM, respectively) and partly, but significantly, recovered with TIG co-treatment (BTZ vs. BTZ + TIG 50 µM: *p* = 0.0114 and *p* = 0.0443 and in KMS20 and KMS28BM, respectively) (Figure 7d).

**Figure 8:**
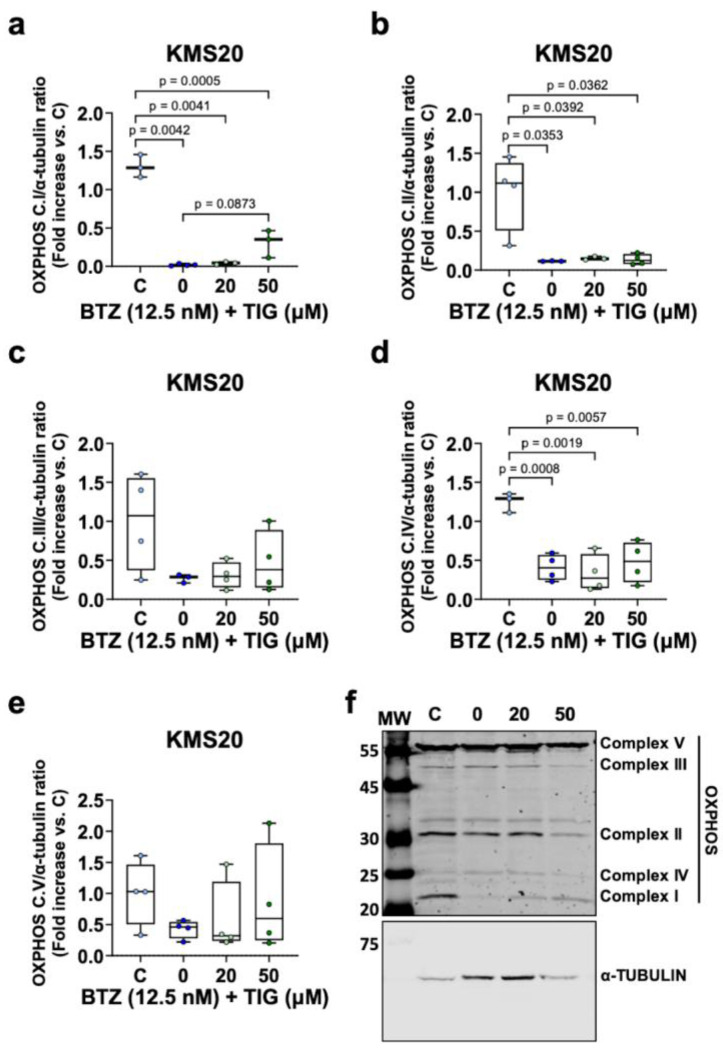
Evaluation of oxidative phosphorylation complexes in KMS20 cells shows a reduction under BTZ treatment. Western blot analysis of mitochondrial respiratory chain complexes, from complex I to complex V, in KMS20 cells (n = 4). (**a**–**e**) Histograms displaying the protein/α-TUBULIN ratio upon quantification of band intensities from control (vehicle-treated cells, C) (n = 3–4), BTZ EC50 (0) (n = 3–4), BTZ EC 50 + 20 µM TIG (20) (n = 3–4), and BTZ + 50 µM TIG (50) (n = 4). Results are expressed using box and whiskers diagrams, where lines represent the median value, boxes the 25th and 75th percentiles, and whiskers mark maximum and minimum values. Significant *p*-values are shown above the boxes. (**f**) Representative western blot analysis of mitochondrial respiratory chain complexes, from complex I to complex V, in KMS20 cells (n = 4) (α-TUBULIN used as loading control). MW: molecular weight marker.

To further understand mitochondrial changes in BTZ-resistant cells treated with these drugs, we assessed the ETC complexes by western blot in KMS20 cells. We observed a reduction in all the complexes under BTZ treatment, reaching statistical significance for complexes I, II, and IV (Figure 8a,b,d, respectively). The addition of TIG to the cell cultures did not significantly change the complex levels (Figure 8a).

In summary, these data indicate that the combination of TIG and BTZ can modify mitochondrial homeostasis and *MYC* expression with a cell type impact in these effects.

### 2.7. TIG and BTZ Combination versus BTZ Single Treatment Improves Survival of Plasma Cells from Patients with Plasma Cell Neoplasms

To further support our findings, we prospectively collected 11 bone marrow samples from 10 patients consecutively studied in our center with plasma cell neoplasm, excluding monoclonal gammopathy of undetermined significance (Table 1).

First, we studied one sample from a patient diagnosed with solitary plasmacytoma of bone and seven from patients newly diagnosed with myeloma, either smoldering (2) or symptomatic (5), according to the IMWG myeloma defining events (Table 1). The patient with solitary plasmacytoma was studied again after evolving to MM 3 months and 2 weeks later. This case had been treated with local radiotherapy for the plasmacytoma. None of these seven patients had received any other therapy against plasma cell neoplasms.

**Figure 9:**
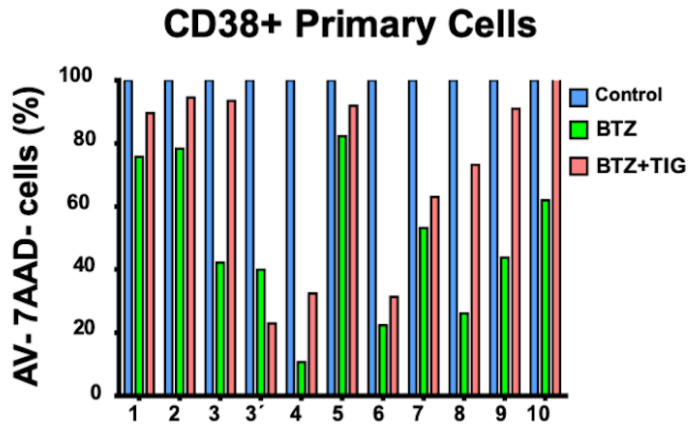
In primary human plasma cell neoplasms TIG-BTZ association partly counteracts BTZ caused viability reduction. Percentage of viable primary plasma cells defined as annexin V (AV) and 7ADD double-negative, CD38-positive cells. Control: AV-/7ADD-, CD38+ cells (blue bars), treated with DMSO vehicle without drugs for 48 h, was set to 100%. Viable CD38+ cells after culturing for 48h with either 12.5 nM bortezomib (BTZ) or BTZ plus 50 µM tigecycline (TIG) (green and pink bars, respectively); the means of two replicas were referred to control. The proportion of viable primary plasma cells was higher after incubating with TIG plus BTZ than after culturing with BTZ, with the exception of patient 3 at the time of progressing to MM (3′) 15 weeks after plasmacytoma diagnosis (3). The patient number corresponds to Table 1.

The incubation of bone marrow mononuclear cells for 48 h with 12.5 nM BTZ (the EC50 for BTZ-resistant KMS20 cells) reduced cell viability, revealed by annexin V and 7ADD double-negativity, in a variable proportion in all samples (Figure 9 and Figure S8). However, when cells were cultured with this concentration of BTZ plus 50 µM TIG, all of them showed a higher proportion of viable cells, excluding the cells from the patient who previously had a solitary plasmacytoma, at the MM diagnosis stage (patient 3′, Figure 9). This might be related to the more aggressive biology of this disease. This prompted us to study three additional MM patients who relapsed during the study. All three had been previously treated with BTZ (Table 1). However, cell viability was reduced with BTZ and recovered in a great proportion with the combined treatment.

In summary, plasma cells of 10 out of 11 samples from 10 patients with malignant plasma cell neoplasms, representative of a wide spectrum of the disease presentation, evidenced a viability raise when incubated with both TIG and BTZ compared to BTZ single treatment.

## 3. Discussion

We observed that the association of the antibiotic TIG, which prevents mitochondrial translation, to the proteasome inhibitor BTZ reprograms cell metabolism and cell cycle, inducing a resistance to BTZ effects.

**Figure 10:**
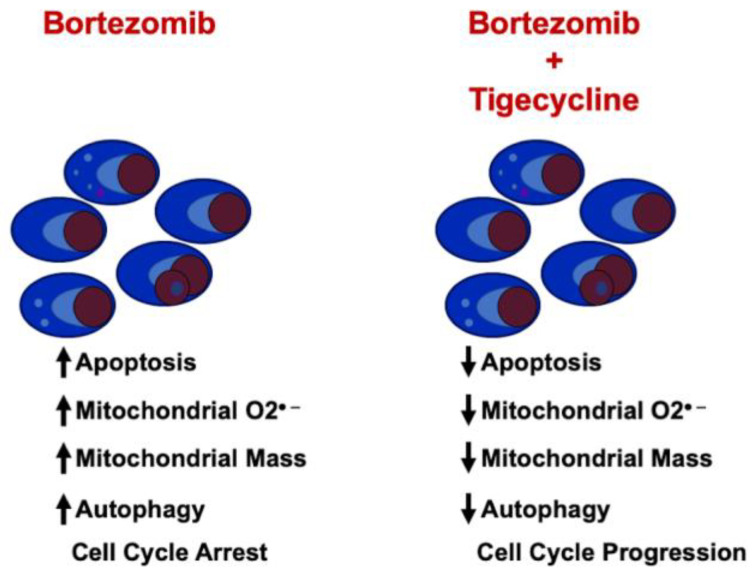
Summarized model of the effect of BTZ and TIG of MM cells. O_2_^•^: superoxide.

In our models, the combination of BTZ and TIG prevents MM cell apoptosis both in cell lines and primary cells. This effect was associated to a decrease in mitochondrial ROS production and changes in mitochondrial functional parameters. Also, the different points of cell cycle arrest of each drug were counterbalanced, and BTZ induced autophagy and mitochondrial mass increase were reverted, when both drugs were combined (Figure 10).

TIG as single treatment induced apoptosis in KMS20 and KMS28BM MM cells, in agreement with experiments performed in other cancer cells [34,35,36]. This contrasts with other studies, which may be due to the lower doses used [39], or to the different cell lines studied [32]. This effect can be influenced by the time of exposure to TIG, as well [36]. We also confirmed the previously described proapoptotic effect of BTZ in both KMS20 and KMS28BM cells, which are more resistant or sensitive to BTZ-mediated cell death, respectively [23]. However, when BTZ was associated to increasing doses of TIG, we observed a TIG dose-dependent reduction of cell death compared to BTZ single treatment effect in both cell lines. Lower concentrations of TIG in association with BTZ EC50 were needed to induce significant changes in apoptosis in KMS28BM compared to KMS20 cells, suggesting that intrinsic cell characteristics influence this effect.

This unexpected result prompted us to gain insight into the mechanisms involved in this process. Cancer cells with higher mitochondrial mass are prone to apoptosis [38]. Consistently, TIG dose-dependently counteracted BTZ-linked increase in mitochondrial mass.

Cell cycle deregulation is a MM hallmark. Therefore, targeting cell cycle progression is of great therapeutic interest [19]. In agreement with previous descriptions [30,32,41], we confirmed in KMS20 cells that BTZ and TIG arrested the cell cycle at G2/M and G0/G1, respectively. Supporting the lack of synergy between TIG and BTZ, we found that the treatment combination significantly reduced the percentage of cells in G2/M cell cycle phase, opposing BTZ effect, and produced a tendency to increase cells in S phase in both cell lines. These findings suggest that the combination of TIG and BTZ neutralizes the cell cycle arrest induced by each drug independently and allows the cells to further progress within the cell cycle.

Single treatment with TIG has been shown to induce autophagy in MM cells through regulation of the AMPK-mTOR pathway [32]. Nevertheless, our data are consistent with a reduction in BTZ-induced autophagy when TIG is associated to this drug. Some drugs can induce both autophagy and apoptosis [17], indicating that both processes are not mutually exclusive. Autophagy can be used to eliminate and recycle old and dysfunctional cell components, helping cell survival, but can also produce autophagy-dependent cell death [48,49]. Autophagy has also been related to these apparently paradoxical processes in MM [49]. It contributes to malignant plasma cell survival [10,16]. However, excessive autophagy has been associated to cell death in MM plasma cells [18]. BTZ increases autophagy and its inhibition can antagonize BTZ cytotoxicity, suggesting that autophagic cell death contributes to BTZ antimyeloma effect [18,49]. Our data show that TIG counteracts BTZ effect on autophagy, as shown by the neutralization of BTZ-induced increase in autophagy markers by the combination with TIG. This was the case of MAP1LC3B-II protein level, which has been closely correlated with the autophagosomes number. As previously recommended, MAP1LC3B-II was compared between the different conditions, rather than its ratio with MAP1LC3B-I, which, typically, is poorly detected [50]. In addition to MAP1LC3B-II, the analysis of autophagic vacuoles, *MAP1LC3B* mRNA and *ULK1* transcript levels showed consistent results. Thus, the combination of TIG with BTZ may oppose to BTZ-induced autophagic cell death, contributing to increased survival.

Of particular interest is the degradation of mitochondria by autophagy, or mitophagy. Elimination of damaged mitochondria through mitophagy depends on the recruitment of Parkin RBR E3 Ubiquitin Protein Ligase (PRKN) to the mitochondria outer membrane by MFN2 and their PINK-dependent binding [43]. Thus, the increase in both *MFN2* and *PINK* expression by BTZ and the reduction that follows upon TIG plus BTZ treatment suggests that the enhancement of mitophagy produced by BTZ is also counteracted by TIG. The TIG-related reduction in *POLRMT* expression, and hence mtDNA replication and transcription in the cells treated with both drugs, supports that mitochondrial biogenesis is also decreased. The reduction in mitochondrial mass may be related to the predominance of this effect over the lessened mitophagy.

High ROS level can trigger cell death of cancer cells [51], including MM cells [52]. Thus, an important mechanism mediating BTZ cytotoxicity in MM is oxidative stress [10]. We confirmed that BTZ increases mitochondrial superoxide levels in our experimental model. Accordingly, the combination of BTZ and TIG reduced the amount of superoxide in association with the reduction in apoptosis observed in this situation. Previous observations have shown that ROS inhibitor VAS3947 also produces MM plasma cell death, but does not synergize with BTZ [12], illustrating the complexity of cell cytotoxicity in which many factors are present and might counteract each other.

The increased mitochondrial ROS production induced by BTZ, and its reduction when combined with TIG, can also partially explain the changes in mitochondrial mass that we observed, as ROS induce mitochondrial permeability transition pore opening that causes mitochondrial swelling and cell death [53,54].

Accumulation of ROS, particularly superoxide and hydrogen peroxide produced by mitochondria, has an essential role in the activation of autophagy [55]. In keeping with this, we observed that, when increasing concentrations of TIG were combined with BTZ, the reduction in autophagy was associated to decreasing mitochondrial superoxide levels. Further supporting our findings, blockage of ROS has been shown to abolish both autophagy and apoptosis in human MM RPMI8266 cells [17].

This might contrast with the induction of mitochondrial ROS by TIG single-drug exposure found in other experimental conditions [42]. Nevertheless, this effect seems to be context dependent with no change in ROS production in some cell types [56], particularly in glycolytic ones compared to cells with high OXPHOS [57].

Elevated ROS in MM cells yields the increase of antioxidants including SOD1 and SOD2 as a balancing mechanism. Also, in relation with BTZ induction of apoptosis through ROS generation, antioxidants can induce BTZ resistance [10,11,12,23,48,58]. Accordingly, we observed a parallel increase in superoxide and superoxide scavengers SOD1 and SOD2, and the key regulator of antioxidant response NFE2L2 in BTZ-treated cells. At the same time, mitochondrial superoxide reduction induced by the TIG and BTZ combination was associated to a downregulation of *SOD1* and *NFE2L2*, and a reduction in SOD2 protein levels. Thus, the decrease in mitochondrial superoxide might be generated by a reduced production rather than an increased degradation. SOD1 has complex functions regulating antioxidant response and repressing mitochondrial respiration [8]. Also, overexpression of mitochondrial SOD2 can produce a metabolic switch from mitochondrial respiration to glycolysis [9]. Thus, reduction of SOD levels might influence the changes in mitochondrial function we observed when the cells are treated with both TIG and BTZ compared to BTZ single treatment.

SOD enzymes catalyze the conversion of superoxide anion into H_2_O_2_. However, H_2_O_2_ raised when BTZ was associated to high TIG concentration. H_2_O_2_ is not only produced in mitochondria. In fact, in MM is mainly generated at the endoplasmic reticulum in the process of immunoglobulin synthesis [10]. In this sense, it is important to consider that the only *PRDX* consistently upregulated in both MM cell lines with TIG plus BTZ combination was *PRDX4*, which is located at the endoplasmic reticulum [10]. PRDX4 levels increase as cells became competent to synthesize immunoglobulin light chains [59]. Thus, high *PRDX4* and H_2_O_2_ levels might reflect an elevated immunoglobulin production of more vigorous neoplastic plasma cells. Supporting this, it has been shown that myeloma cells exposed to H_2_O_2_ in culture increase antiapoptotic BCL2 protein levels [60], suggesting that at certain levels of it might have a role in promoting MM cell survival.

In contrast, the levels of cytosolic PRDX1 are less clearly related to B cell maturation [59]. In KMS20, we observed that *PRDX1* expression is reduced in BTZ treated cells, and recovered up to control levels after TIG treatment, although no change was detected in KMS28BM cells. This suggests that the expression of *PRDX1* and other antioxidant genes, such as *TXN* and *CAT*, is influenced by the intrinsic characteristics of the cell line. PRDX1 in particular is of special interest as it can interact with MYC to module its functions and produce a shift in ROS peak levels from nuclear to cytoplasmic localization [61]. This might account for some of the differences observed between both cell lines. Conversely, *PRDX5*, located at the cytosol, mitochondria, and nucleus [10], was downregulated in both cell lines upon incubation with either BTZ or all BTZ+TIG combinations. Therefore, this is a BTZ related effect that is not modified by TIG. Other antimyeloma drugs have similar effect on *PRDX5* [62].

The case of *TXN* is particularly striking, as it followed an inverse response to the drugs in both cell lines. Besides the antioxidant activity, *TXN* is anti-apoptotic and increases cancer cell proliferation. Part of its function is achieved through translocation from the cytoplasm to the nucleus, where it regulates the expression of several transcription factors [63]. Increased levels of TXN have been associated to MM cells’ viability and BTZ resistance [64,65]. Thus, BTZ-resistant KMS20 may rely more on TXN as a mechanism to promote cell survival than BTZ-sensitive KMS28BM cells. This might explain in part that in KMS20 cells *TXN* is downregulated with BTZ incubation compared to the control levels and upregulated when TIG is associated to BTZ, helping to counteract BTZ proapoptotic effect. Instead, in KMS28BM cells *TXN* followed the opposite expression pattern, replicating the behavior of other antioxidants.

It has been shown that G6PD overexpression can favor MM cell proliferation, which was linked to ROS levels reduction, and G6PD knockdown reduced cell survival [45]. In contrast, we observed that in MM cells co-cultured with TIG and BTZ *G6PD* expression decreased compared to cells treated only with BTZ, while survival increased. G6PD is a limiting enzyme of the pentose phosphate pathway, which is a source of antioxidant power, but it is also an alternative metabolic route to glycolysis and OXPHOS. Therefore, the changes we observed under the combined treatment may be related to the reduction of mitochondrial oxidative stress and to modifications in cell metabolism.

The presented data point to mitochondria as a central actor in our observations. POLRMT is involved in mtDNA transcription and replication [46]. In agreement with the reduction in mitochondrial mass produced by TIG plus BTZ combination, *POLRMT* transcript levels also decreased. However, the mRNA amount of the factors related to transcription initiation *TFAM* and *TFB1M* increased. This may be part of a mechanism to compensate the reduction of POLRMT to maintain mtDNA transcription. Accordingly, in previous studies *POLRMT* knockdown associated to an increase in *TFAM* and a tendency to elevate *TFB1M* mRNAs, together with normal levels of ETC protein levels, including mitochondrially encoded MT-CO1 (COX1) and MT-CO2 (COX2) [46]. Additionally, *POLRMT, TFAM, TFB2M*, and *TFB1M* have different relative expression levels among several tissues and cellular growing conditions [47]. This might be related to their particular functions. So, TFAM is not only involved in mtDNA transcription and replication, but also in mtDNA packaging, [66] and in damaged DNA degradation [67]. This explains that it has been related both to improved cell survival and mitochondrial function [66]. TFB1M also favors mitochondrial function. It is thought to predominantly regulate translation of mitochondrial genes, rather than transcription, by demethylating 12S ribosomal RNA, which is required for the assembly of mitochondrial ribosomes. Consistently, TFB1M-deficient cells show reduced levels of ETC proteins without affecting their transcription and decreased ATP production [68,69]. Thus, *TFB1M* increase may be a mechanism for compensating TIG-mediated inhibition of mitochondrial translation.

BTZ resistance has been related to increased OXPHOS and improved mitochondrial function parameters, including higher OCR, mitochondrial ATP, and *ATP5B* mRNA levels, and lower superoxide [23,24,25,26]. In keeping with this, BTZ-resistant KMS20 reduced OCR upon BTZ treatment and slightly increased it when TIG was added to BTZ, while it is reduced in BTZ sensitive KMS28BM. This suggests that TIG addition to BTZ can partly restore BTZ-induced OXPHOS impairment and help to accomplish BTZ resistance in KMS20 cell line. In agreement with this, we observed a reduction of all ETC complexes in KMS20 cells under BTZ treatment and a tendency to increase in the combined treatment. The inhibition of ETC complexes has been previously related to BTZ resistance in lung cancer and MM cells [27,28]. This produced a reduction in OCR and an ECAR increase [28], both found reduced in our experimental conditions under BTZ treatment in this cell line. Different metabolic requirements and roles of mitochondria according to the experimental settings including cell types can be at the origin of these observations.

A reduced mitochondrial mass and a more efficient mitochondrial function would contribute to mitochondrial superoxide reduction. Hence, in that situation, high levels of superoxide antioxidants would no longer be needed [10], leading to their decrease.

To further clarify the role of TIG in the context of BTZ treatment, we studied in vitro cells from a representative group of patients with malignant plasma cell neoplasms. MGUS was excluded, as it is a low-aggressive precursor/premalignant condition that rarely requires treatment. The samples collected corresponded to two quiescent myelomas, a bone plasmacytoma, five patients at the time of diagnosis of active MM, and three patients in MM relapse after receiving different treatments, but the three of them exposed to BTZ.

An enhancement between BTZ and TIG in terms of the production of apoptosis was observed only in one sample from a patient with symptomatic MM after a rapid evolution from solitary plasmacytoma, with the appearance of multiple bone lesions, noteworthy elevation of beta-2 microglobulin, LDH increase, anemia, renal involvement, and fast-onset hypercalcemia. Therefore, TIG association to BTZ might be beneficial in cases with evolved disease and some patients with BTZ resistance. Nevertheless, we did not observe a potentiation between the two drugs, but an increase in viable cells, in relapsed patients who had previously undergone treatment with BTZ (in vivo).

In contrast to our findings, previous research focused on human plasma cell leukemia JJN3 cell line in vitro studies and in vivo-generated xenografts has claimed synergistic effects between TIG and BTZ [33]. This cell line was selected due to the high level of *MYC* expression, mtDNA copy number, and cytochrome C oxidase activity among four other MM cell lines of human origin. Also, JJN3 studies were performed with a single low concentration of TIG (3.7 µM). This, together with cell line differences, may account for the apparent discrepancy between both studies. In fact, the combination of TIG and BTZ in JJN3 cells reduced MYC levels. This MYC downregulation was considered to be a major factor in the origin of their observations. However, we have observed that TIG addition to BTZ increased *MYC* expression, although its level was dependent on the cell line and was linked to high TIG concentrations. Proto-oncogene MYC has been related to multiple functions, such as enhancing transcription, cell cycle progression, ribosome biogenesis, and glycolysis [20,21]. MYC also regulates cellular proliferation and apoptosis, with a different effect depending on its levels and the cell context [20]. Likewise, MYC overexpression has been related to ROS increase, but levels closer to physiological expression reduce ROS [70]. MYC-dependent ROS reduction has been associated to *NFE2L2* upregulation, which we did not observe, but other mechanisms may be involved including the induction of glutathione synthesis. In plasma cell neoplasms MYC has been associated to disease progression from MGUS to MM and to a shorter survival of MM patients [20]. Thus, *MYC* upregulation may explain part of our data. MYC expression has been reported to be higher in KMS28BM than in KMS20 cells [71], and we observed that *MYC* expression raised in KMS28BM with lower TIG concentrations combined with BTZ EC50 than in KMS20 cells. Consequently, MYC levels may also account for some of the differences found in both cell lines.

On the other hand, JJN3 cells showed reduced levels of the mitochondrial respiratory chain complex I component NDUFB8 and complex IV protein MT-CO2, together with cytochrome C oxidase activity with the combined treatment. Nevertheless, this effect was not clearly shown in BTZ-resistant JJN3 BR cells, suggesting that mitochondrial respiration can be different in BTZ-sensitive and resistant cells, as we observed.

In addition, low TIG (3.7 µM) and BTZ (2 nM) concentrations were assayed on primary cells from three MM samples and one MGUS, showing a reduction in plasma cell proportion and a minor decrease in mitochondrial membrane potential. Again, the dose of the drugs and the different assays performed, together with the low number of MM primary cells assessed, may justify the different conclusions reached in both studies. Interestingly, in the JJN3 cell xenograft model, mice treated only with TIG had a longer survival than those treated with BTZ plus TIG, suggesting that the combination is less effective for treating myeloma than TIG alone in this model [33].

Even so, the association of both drugs could be positive in some patients with very aggressive disease, as suggested by our study and Ortiz-Ruiz A et al. [33], while in all other cases, it could be ineffective or even counterproductive. These findings may be relevant not only to be careful if considering MM treatment with the association of these drugs, but also when TIG is used as an antibiotic in MM patients treated with BTZ and potentially with other proteasome inhibitors.

Finally, this study points to the need for personalized treatments, the importance of drug resistance, and the involvement of cell metabolism in treatment response.

## 4. Materials and Methods

### 4.1. Cell Lines and Cultures

Cell lines KMS20 (JCRB1196) and KMS28BM (JCRB1192) were obtained from the Japanese Collection of Research Bioresources Cell Bank (Ibaraki city, Osaka, Japan).

Cells were grown in suspension at 37 °C and 5% CO_2_ with RPMI-1640 supplemented with 10% (*v*/*v*) of FBS; 2 µmol/L L-glutamine; 100 U/mL penicillin G; and 100 µg/mL streptomycin (all of them from Gibco, ThermoFisher Scientific, Waltham, MA, USA). Cells were cultured in 6-well plates and exposed to vehicle dimethylsulfoxide (DMSO), BTZ, TIG, or a constant dose of BTZ (12.5 nM for KMS20 and 4.5 nM for KMS28BM) with increasing TIG concentrations (5–60 µM). BTZ and TIG were obtained from Selleck Chemicals (Houston, TX, USA) and dissolved in DMSO to a stock concentration of 100 mmol/L.

### 4.2. Primary Cells

Fresh bone marrow aspirates were obtained in EDTA from patients older than 18 years old with plasma cells neoplasms excluding monoclonal gammopathy of undetermined significance. All cases were subsequently studied at Hospital Clínico San Carlos, had clonal plasma cells over 95% of total in the flow cytometry analysis, and their neoplastic plasma cells expressed CD38 surface marker. All participants signed a written informed consent form, and this study was approved by the Ethics Committee of Hospital Clínico San Carlos (19/305-E_BS).

Mononuclear cells were isolated by Ficoll (Lymphoflot, Bio-Rad, Hercules, CA, USA) density gradient centrifugation at 500 G for 30 min. Cells were cultured as above for 48 h with either vehicle, 4.5 nM BTZ, or 4.5 nM BTZ plus 50 µM TIG.

Plasma cells were identified and gated for following flow cytometry analysis with anti-Human CD38 antibody (BD Biosciences, San Jose, CA, USA).

### 4.3. Flow Cytometry

Cells were cultured to a density of 3 × 10^5^/mL. Two technical replicas per treatment condition and vehicle control were performed, and the averaged values were compared with the data obtained in independent experiments.

Apoptosis was measured by flow cytometry via staining with annexin V and 7-Aminoactinomycin D (7AAD), using PE annexin V Apoptosis Detection Kit (BD Pharmingen, San Diego, CA, USA) according to the protocol provided by the manufacturer.

Cell cycle was analyzed using APC BrdU Flow kit (BD Pharmingen) by measuring the incorporation of 5-bromo-2-desoxyuridine, a thymidine analogue, and DNA staining with 7-AAD; this procedure was performed following the manufacturer’s instructions.

Autophagy analysis: The difference in autophagic flow between the different treatments was assessed by measuring the levels of monodansylcadaverine, a molecule attached to autophagic vesicles, using Autophagy Assay Kit ab139484 (Abcam, Cambridge, UK). Cells were collected by centrifugation and treated with stain for 30 min at 37 °C. A positive control using 500 nM Rapamycin was included. The solution was analyzed with FITC-A channel.

The measurement of mitochondrial mass was performed as before [53]. Briefly, myeloma cells were incubated at 37 °C for 30 min in presence of 200 nM MitoTracker Green FM (Invitrogen-ThermoFisher, Waltham, MA, USA) and the staining was visualized with FITC-A channel.

Mitochondrial ROS study was also carried out as previously [53]. Cells were incubated for 15 min 2 µM MitoSOX (Invitrogen-ThermoFisher) for superoxide radicals analysis and assessed with ECD-A channel.

To study intracellular hydrogen peroxide levels, cells were stained at 37 °C for 30 min with Cell Meter Intracellular Fluorimetric Hydrogen Peroxide *Blue Fluorescence Optimized for Flow Cytometry* (AAT Bioquest, Las Positas, CA, USA), following the manufacturer’s protocol.

Flow cytometry data were acquired using CytoFlex (Beckman Coulter, Brea, CA, USA) and were analyzed using Cytexpert 2.6 (Beckman Coulter) and Kaluza 2.2.1 (Beckman Coulter).

### 4.4. Gene Expression Studies

Gene expression was studied as before [53]. Briefly, RNA was extracted with TRI reagent (Sigma-Aldrich, St. Louis, MO, USA) according to manufacturer specifications. RNA concentration was quantified by spectrophotometry with a nanodrop equipment (Thermo Fisher, Waltham, MA USA) and 1 µg was retrotranscribed with SuperScript IV Reverse Transcriptase using random hexamers and following manufacturer recommendations (Invitrogen-Thermo Fisher).

Real-time PCR was performed with 10 ng cDNA for each assay. Expression assays (ThermoFisher-ABI) were the following: AKT serine/threonine kinase 1 (*AKT1*) Hs00178289_m1; ATP synthase F1 subunit beta (*ATP5B*) Hs00969569_m1; catalase (*CAT*) Hs00156308_m1; glucose-6-phosphate dehydrogenase (*G6PD*) Hs00166169_m1; glutathione peroxidase 1 (*GPX1*) Hs00829989_gH; microtubule associated protein 1 light chain 3 beta (*MAP1LC3B*) Hs00917682_m1; mitofusin 2 (*MFN2*) Hs00208382_m1; mechanistic target of rapamycin kinase (*MTOR*) Hs00234508_m1; MYC oncogene proto-oncogene bHLH transcription factor (*MYC*) (Hs00153408_m1); NFE2-like bZIP transcription factor 2 (*NFE2L2*) Hs00232352_m1; OPA1 mitochondrial dynamin-like GTPase (*OPA1*) Hs01047013_m1; phosphatase and tensin homolog-induced kinase 1 (*PINK1*, Hs00260868_m1); RNA polymerase mitochondrial (*POLRMT*) Hs04187596_g1; Peroxiredoxin 1 (*PRDX1*) Hs00602020_mH; Peroxiredoxin 4 (*PRDX4*) Hs01056076_m1; Peroxiredoxin 5 (*PRDX5*) Hs00201536_m1; superoxide dismutase 1 (*SOD1*) Hs00167309_m1; superoxide dismutase 2 (*SOD2*) Hs00167309_m1; transcription factor A, mitochondrial (*TFAM*) Hs01082775_m1; transcription factor B1, mitochondrial (*TFB1M*) Hs01084404_m1; thioredoxin (*TXN*) Hs00828652_m1; thioredoxin 2 (*TXN2*) Hs00429399_g1; and unc-51-like autophagy activating kinase 1 (*ULK1*) Hs00177504_m1.

The quantification was related to control TATA-box binding protein (*TBP*) Hs99999910_m1 and ribosomal protein L30 (*RPL30*) Hs00265497_m1 (ThermoFisher-ABI), using TaqMan™ Fast Universal PCR Master Mix (2X) in an ABI 7500 Fast Real-time PCR system (Thermo Fisher) and analyzed using the 2^−ΔΔCT^ method. Reference genes were tested with NormFinder 21 software [72], Figure S9.

### 4.5. Western Blotting

Cells were seeded to a density of 6 × 10^5^ cells/mL, collected by centrifugation after treatment, and lysed in ice for 20 min with lysis buffer containing 0.5% NP-40 (*v*/*v*), 1 mM Tris-HCl pH = 6.8, 150 nM NaCl, and Protease Inhibitor Cocktail EDTA-free (Roche, Basel, Switzerland). Protein concentration was measured with Bradford Reagent (Sigma-Aldrich).

For MAP1LC3B and Mn-SOD (SOD2) study, 40 µg of protein samples were diluted in 4X SDS-PAGE Sample Buffer (1M Tris-HCl, pH = 6.8; 10% SDS; 0.1 M DTT; 10% glycerol; H_2_O and bromophenol blue). Protein electrophoresis was performed using a 4–20% gradient gel (Bio-Rad). After semi dry transfer, membranes were blocked with 5% skimmed milk in Tween–Tris-buffered saline (TTBS): 10 mM Tris-HCl, pH 7.4, 150 mM NaCl, and 0.05% Tween-20. PVDF membranes were incubated with MAP1LC3B (3868, Cell Signaling Technology, Danvers, MA, USA) and Mn-SOD (06–984, Merck, Darmstadt, Germany) primary antibodies diluted in Tween–Tris-buffered saline (TTBS) with 5% BSA and 0.02% sodium azide. Primary antibodies were immunodetected using anti-Rabbit HRP-conjugated secondary antibody (1:5000 in TTBS, 12-348, Merck, Darmstadt, Germany). Proteins were visualized by incubating with Pierce ECL Western Blotting Substrate (32209, Thermo Fisher Scientific, Waltham, MA, USA) and visualized using the VWR Imager Chemi Premium documentation system (VWR, Radnor, PA, USA). Loading was normalized with β-Actin (8457, Cell Signaling Technology, Danvers, MA, USA) and signal intensity was determined by densitometric analysis of each band using ImageJ Fiji win64 software [73].

To evaluate oxidative phosphorylation protein complexes, 15 µg of protein was diluted in 2X SDS-PAGE sample buffer (100 mm Tris/HCl, pH 6.8, 3% SDS, 1 mM EDTA, 2% 2-β-mercaptoethanol, and 5% glycerol). PVDF membranes were incubated with OXPHOS (#45-8199; 1:500; Invitrogen, Waltham, MA, USA) and α-TUBULIN (#600-401-880; 1:5000; Thermo Fisher Scientific, Waltham, MA, USA) primary antibodies diluted in Tween–Tris-buffered saline (TTBS) with 5% BSA and 0.02% sodium azide. Primary antibodies were immunodetected using horseradish peroxidase-conjugated anti-rabbit (1:10,000 in TTBS with 0.01% SDS; 925-32213, LI-COR Biosciences, Lincoln, NE, USA) or anti-mouse secondary antibody (1:10,000 in TTBS with 0.01% SDS; 925-68070, LI-COR Biosciences, Lincoln, NE, USA). Loading was normalized with α-TUBULIN and band intensities were quantified using Image StudioTM Lite Software v5.2. (LI-COR Biosciences, Lincoln, NE, USA).

### 4.6. Seahorse Analysis

Oxygen consumption rate (OCR) and extracellular acidification rate (ECAR) were measured in triplicate on 2 × 10^5^ cells with a Cell Mito Stress Test kit (Agilent Technologies, Santa Clara, CA, USA), following the manufacturer’s instructions. Briefly, cells were treated over 60 min with mitochondrial oxidative phosphorylation (OXPHOS)-selective inhibitors: 1.5 μM of oligomycin, an inhibitor of the ATP synthase; 0.5 μM carbonylcyanide-*p*-trifluoromethoxyphenylhydrazone, a mitochondrial OXPHOS uncoupler; and a cocktail of rotenone and antimycin A at 0.5 μM, inhibitors of complex I and III from the respiratory chain. OCR and ECAR were registered during the run, allowing for assessment of mitochondrial performance and glycolytic function, respectively. Agilent Seahorse Wave was used to analyze data and files from Seahorse XFp analyzer (Agilent, Santa Clara, CA, USA).

### 4.7. Statistical Analysis

The data are presented as mean and standard error of the mean (SEM) and were analyzed by one-way ANOVA followed by Tukey’s test to compare more than two groups of data and the Mann–Whitney U test for two groups, using GraphPad Prism 9.0.2 (San Diego, CA, USA). Differences between groups were considered significant when *p* < 0.05. Half-maximal effective concentration (EC50) was also calculated using GraphPad Prism 9.0.2.

For western blot analysis, the normality of the variables was assessed with the Shapiro–Wilk test (less than 30 data). Outliers were detected and discarded with Grubbs’ test (https://www.graphpad.com/quickcalcs/Grubbs1.cfm, GraphPad online tool accessed on 7 February 2024). Statistical significance was assessed using unpaired two-tailed Student’s *t-*tests and unpaired non-parametric Mann–Whitney U tests. For unequal variances after the Student’s *t-*test, Welch’s *t-*test was applied.

## Figures and Tables

**Table 1:** Main characteristics of patients.

	P1	P2	P3	P3’	P4	P5	P6	P7	P8	P9	P10
**Disease stage**	D SMM	D SMM	D Plasmac	D MM	D MM	D MM	D MM	D MM	R MM	R MM	R MM
**Age (years)**	82	63	55	55	47	69	87	79	77	72	81
**Sex**	M	F	M	M	F	F	M	M	F	F	F
**S M-spike (g/dL)**	1.49	1.62	0.83	2.10	0.13	2.38	2.36	3.97	1.65	UD	0.56
**Type of Ig (serum)**	IGG L	IGG K	IGA K	IGA K	IGG L	IGA L	IGA K	IGG K	IGG K	K	IGA L
**FLC K/L ratio**	0.03	26.2	40.04	1240.74	0.00237	0.03	1.99	41.36	57.88	12.33	0.02
**Urine M-spike (mg/24 h)**	322.6	IFix+	IFix+	1101.1	4910.4	251.7	90.4	3.7	UD	UD	434.7
**% PC (cytology)**	10–15	5–30	5–7	5–100	60–70	70–90	50–70	60–70	20–60	40–75	5–10
**IF. CPC %**	98.15	99.5	96.3	99.4	99.03	99.9	99.9	98.84	96.45	99.66	96.2
**FISH**	NA	NA	NA	FISH-	NA	NA	FISH-	FISH-	FISH-	NA	+1q21 [70/100] *
**S creatinine (mg/dL)**	1.39	0.69	0.45	2.19	0.98	1.02	1.82	0.87	0.62	1.41	1.49
**S. calcium (mg/dL)**	9.8	9.7	9.3	13.9	9.7	9.3	9	9.4	8.4	11.7	9.8
**Hb (g/L)**	128	118	152	80	80	91	123	120	110	105	122
**S. albumin (g/dL)**	3.8	3.8	3.8	2.2	4.6	3.1	3.5	3.9	3.5	4.5	3.6
**LDH (U/L)**	267	421	181	292	575	452	313	264	193	236	491
**S. B2m (mg/L)**	5.1	2.1	2.4	10.1	6.1	5.3	5	2.9	3	NA	7.5
**Bone lytic lesions**	No	Yes	Yes, single	Yes, multiple	Yes	Yes	NA	No	Yes (old)	Yes	Yes
**Treatment**	No	No	No	RT	No	No	No	No	8xVMP 22xRd	6xRVd Plus ASCT	9xVMP 6xVMP

Ten patients were included in the study, 3 and 3′ correspond to the same patient analyzed with solitary plasmacytoma and MM, respectively. * Number of CD138 positive cells with 1q21 extra copy over total CD138 positive cells. *p*: patient, D: newly diagnosed; SMM: smoldering multiple myeloma; Plasmac: solitary plasmacytoma; MM: multiple myeloma; R: relapsed; FLC: free light chain; K: kappa; L: lambda; Type of Ig: type of clonal immunoglobulin heavy chain; PC: plasma cells; IF: immunophenotype; CPC: clonal plasma cells; S: serum, Hb: hemoglobin; B2m: beta-2 microglobulin; M: male; F: female; IFix+: detectable by immunofixation; UD: undetectable; FISH-: fluorescence in situ hybridization negative for t(11;14), t(4;14), del(17p13.1), 1p32/1q21. NA: non-analyzed; RT: radiotherapy; D-RVd: daratumumab, lenalidomide, bortezomib, and dexamethasone; VMP: bortezomib, melphalan, and prednisone; Rd: lenalidomide and dexamethasone; RVd: lenalidomide, bortezomib, and dexamethasone; ASCT: autologous stem cell transplant; D-VMP: daratumumab, bortezomib, melphalan, and prednisone.

## Data Availability

The data presented in this study are available on request from the corresponding author.

## Supplementary Materials

The following supporting information can be downloaded at: https://www.mdpi.com/article/10.3390/ijms25094887/s1.

## Abbreviations

7AAD (7-Aminoactinomycin D), BTZ (bortezomib), DMSO (dimethylsulfoxide), ECAR (extracellular acidification rate), EC50 (effective concentration 50), MM (multiple myeloma), MGUS (monoclonal gammopathy of undetermined significance), OCR (oxygen consumption rate), OXPHOS (mitochondrial oxidative phosphorylation), ROS (reactive oxygen species), TIG (tigecycline).
