# Supplementary material for: Tigecycline Opposes Bortezomib Effect on Myeloma Cells Decreasing Mitochondrial Reactive Oxygen Species Production
Source: Int J Mol Sci. 2024 Apr 30;25(9):4887. doi: 10.3390/ijms25094887 (PMC11084384; doi:10.3390/ijms25094887)
Supplement: Supplementary file 1 [file ijms-25-04887-s001.zip › Supplementary Figures Legends.pdf]

SUPPLEMENTARY FIGURES LEGENDS

Supplementary Figure S1. Representative flow cytometry studies of cell viability and apoptosis of KMS20 and KMS28BM cell lines. (**a**) KMS20 cells analysis: AV/7AAD double negative cells (viable cells) are shown in blue, AV positive/7AAD negative events (early apoptotic cells) appear in green and AV/7AAD double positive cells are depicted in pink; for simplicity only control (DMSO), BTZ EC50 and BTZ EC50 plus the highest concentration of TIG after 48 hours incubation are shown. (**b**) Representative flow cytometry experiment on KMS28BM cells shown as before.

Supplementary Figure S2. Representative flow cytometry studies of mitochondrial mass and cell cycle. (**a**) Mitochondrial mass studies. On the top, studies on KMS20 cells, for simplicity only control (DMSO), BTZ EC50 and BTZ EC50 plus 50 µM TIG after 48 hours incubation are shown. On the bottom right-hand side, representative studies on KMS28BM cell line as before. (**b**) Cell cycle studies of a TIG plus BTZ combination experiment in KMS20 cells, cells on G0/G1 phase are shown in blue, G2/M in pink and cells on S phase are depicted with orange color. (**c**) Cell cycle studies of a TIG plus BTZ combination experiment in KMS28BM cells, cells on G0/G1 phase are shown in blue, G2/M in orange and cells on S phase are depicted with orange color.

Supplementary Figure S3. Gene expression of autophagy key genes in KMS28BM cell line follow the pattern observed in KMS20 cell line. (**a**) mRNA expression levels *MAP1LC3B* (left) and *ULK1* (right). (**b**) mRNA expression of

mitophagy master genes *PINK1* (left) and *MFN2* (right). In all studies KMS28BM cells were cultivated for 48h. C: DMSO control; 0: BTZ EC50 (4.5 nM); 10: 4.5 nM BTZ plus 10 µM TIG; 20: 4.5 nM BTZ plus 20 µM TIG and 50: 4.5 nM BTZ with 50 µM TIG. RNA was retrotranscribed and quantified by RT-qPCR normalizing with *TBP* expression (*RPL30* normalization had similar results, not shown). Histograms show means ± SEM of 3 independent experiments. *p<0.05.

Supplementary Figure S4. MitoSOX Red flow cytometry analysis. Representative studies of KMS20 and KMS28BM cells, top and bottom panels, respectively, are shown. For simplicity only control (DMSO), BTZ EC50 and BTZ EC50 plus 50 µM TIG after 48 hours incubation are displayed.

Supplementary Figure S5. Gene transcripts of other antioxidants did not match or did not change. (**a**) mRNA expression levels of *SOD2* in KMS20 (left) and KMS28BM (right) cells. The study was performed by RT-qPCR after 48h incubation with either DMSO vehicle (C), the EC50 of BTZ for each cell line (0) or BTZ EC50 plus increasing concentrations of TIG, as shown. *TBP* normalized data are shown, error bars indicate means ± SEM of 3 independent experiments. (**b**) mRNA expression of *TXN*, *TXN2* and *GPX1*, as indicated, quantified by RT-qPCR in KMS20 (top) and KMS28BM (botom) cells as in (a). N= 2-3. *p<0.05, **p<0.01 and ****p<0.0001.

Supplementary Figure S6. *SOD2, AKT*, *MTOR* and *OPA1* expression did not change. mRNA expression of *AKT*, *MTOR* and *OPA1* in KMS20 cells, as indicated. In all studies cells were cultivated for 48h. C, DMSO control; 0, BTZ

EC50 (12.5 and 4.5 nM in KSM20 and KSM28BM, respectively), for the additional bars BTZ EC50 and increasing concentrations of TIG were used as indicated. RNA was retrotranscribed and quantified by RT-qPCR normalizing with *TBP* expression. Histograms show means ± SEM of 3 independent experiments.

Supplementary Figure S7. Basal extracellular acidification rate (ECAR). Study on KMS20 (left) and KMS28BM (right) incubated for 48 hours with either vehicle (C); the EC50 of BTZ or EC50 of BTZ plus tigecycline, as indicated. Plots show means ± SEM of 3 independent experiments, *p<0.05.

Supplementary Figure S8. Representative human primary plasma cell neoplasms studies. (**a**) Gating strategy for flow cytometry analyses of bone marrow primary cells from MM patients followed in this study. SSC, side scatter; FSC, forward scatter. (**b**) Representative flow cytometry analysis on CD38 positive cells gated as in (a), the annexin V (horizontal axis) and 7ADD (vertical axis) study is shown. Staining of control (vehicle) cells, BTZ and BTZ plus TIG treated cells are displayed on the left, central and right panels respectively. The percentage of cells in each quadrant is also indicated.

Supplementary Figure S9. NormFinder software analysis of reference genes. Stability value of control genes *TBP* and RPL30 compared to other genes analyzed in the study. The stability value of both *TBP* and RPL30, 0.027 and RPL30, respectively, was below the cut-off value of 0.15
