# Supplementary material for: Tigecycline Opposes Bortezomib Effect on Myeloma Cells Decreasing Mitochondrial Reactive Oxygen Species Production
Source: Int J Mol Sci. 2024 Apr 30;25(9):4887. doi: 10.3390/ijms25094887 (PMC11084384; doi:10.3390/ijms25094887)
Supplement: Supplementary file 1 [file ijms-25-04887-s001.zip › Table S1.pdf]

PARAMETERS	METHOD	MOLECULAR MARKER	ASSAY SUMMARY	MATERIALS
Cell viability and rate of death	Flow cytometry	Phosphatydilserine *(Annexin V)* DNA *(7AAD)*	Annexin V is measured via PE channel (585-42 nm) and 7AAD is measured via PC5.5 (690-50 nm)	*PE annexin V Apoptosis Detection Kit (BD Pharmingen)*
Cell cycle	Flow cytometry	BrDU *(anti APC-BrDU)* DNA *(7AAD)*	Anti-BrDU fluorophore is measured via APC channel (660-20 nm) and 7AAD is measured via PC5.5 (690-50 nm)	*APC BrdU Flow kit (BD Pharmingen)*
Mitochondrial levels	Flow cytometry	Mitochondrial mass	Live cells are stained with a dye that accumulates in the mitochondria, then measured via FITC channel (516 nm).	*MitoTracker Green FM (Invitrogen)*
Autophagy rate levels	Flow cytometry	Monodansyl cadaverine (MDC)	MDC is marked with a fluorophore and then measured via FITC channel (525-40 nm) Rapamycin is used as a positive control.	*Autophagy Assay Kit ab139484 (Abcam)*
	Western Blot	LC3B	Proteins extracts are obtained with lysis buffer and separated by electrophoresis. Samples are transferred to a PVDF membraned and incubated with antibody. Visualisation is performed using ECL at 16 kDa	*3868 (Cell Signaling Technology)*
	Quantitative Real Time PCR	*MAP1LC3B MFN2 PINK1 ULK1*	cDNA samples were obtained from mRNA by retrotranscription, amplified real time PCR and analysed using the $2^{-\Delta\Delta CT}$ method (RT-qPCR).	*Hs00917682_m1 Hs00208382_m1 Hs00260868_m1 Hs00177504_m1 (ThermoFisher)*
Oxidative mitochondrial state	Flow cytometry	Superoxide radicals in the mitochondria	MitoSOX Red oxidises with mitochondrial superoxide and the fluorescence is measured via ECD channel (610 nm).	*MitoSOX (Invitrogen)*
	Western Blot	Mn-SOD (SOD2)	Proteins extracts are obtained with lysis buffer and separated by electrophoresis. Samples are transferred to a PVDF membraned and incubated with antibody. Visualisation is performed using ECL at 22 kDa	*06-984 (Merck)*

	Quantitative Real Time PCR	*SOD1*<br>*SOD2*<br>*NFE2L2*<br>*G6PD*	RT-qPCR, as before	*Hs00167309_m1*<br>*Hs00167309_m1*<br>*Hs00232352_m1*<br>*Hs00166169_m1*<br>*(ThermoFisher)*
Cell hydrogen peroxide levels and antioxidants	Flow cytometry	H_2O_2	Cells are stained prior to treatment and then measured via Pacific Blue channel (450 nm)	*Cell Meter Intracellular Fluorimetric Hydrogen Peroxide*<br>*(AAT Bioquest)*
	Quantitative Real Time PCR	*CAT*<br>*GPX1*<br>*PRDX1*<br>*PRDX4*<br>*PRDX5*<br>*TXN*<br>*TXN2*	RT-qPCR, as before	*Hs00156308_m1*<br>*Hs00829989_gH*<br>*Hs00602020_mH*<br>*Hs01056076_m1*<br>*Hs00201536_m1*<br>*Hs00828652_m1*<br>*Hs00429399_g1*<br>*(ThermoFisher)*
Mitochondrial homeostasis and energy production	Quantitative Real Time PCR	*ATP5B*<br>*MYC*<br>*POLRMT*<br>*TFAM*<br>*TFB1M*	RT-qPCR, as before	*Hs00969569_m1*<br>*Hs00153408_m1*<br>*Hs04187596_g1*<br>*Hs01082775_m1*<br>*Hs01084404_m1*<br>*(ThermoFisher)*
	SeaHorse	Complex I<br>*(Rotenone)*<br>Complex III<br>*(Antimycin A)*<br>Complex V<br>*(Oligomycin)*<br>Proton gradient in mitochondrial membranes<br>*(FCCP)*	Cells are treated with modulators of respiration at different time points to measure different aspects of mitochondrial performance	*Agilent SeaHorse*
	Western Blot	Electron Transport Chain (Complex I to V)	Proteins extracts are obtained with lysis buffer and separated by electrophoresis. Samples are transferred to a PVDF membraned and incubated with antibody. Visualisation is performed via fluorescence from 10 to 50 kDa	*OXPHOS*<br>*45-8199 (Invitrogen)*
