# Supplementary figures and images for: Tigecycline Opposes Bortezomib Effect on Myeloma Cells Decreasing Mitochondrial Reactive Oxygen Species Production
Source: Int J Mol Sci. 2024 Apr 30;25(9):4887. doi: 10.3390/ijms25094887 (PMC11084384; doi:10.3390/ijms25094887)

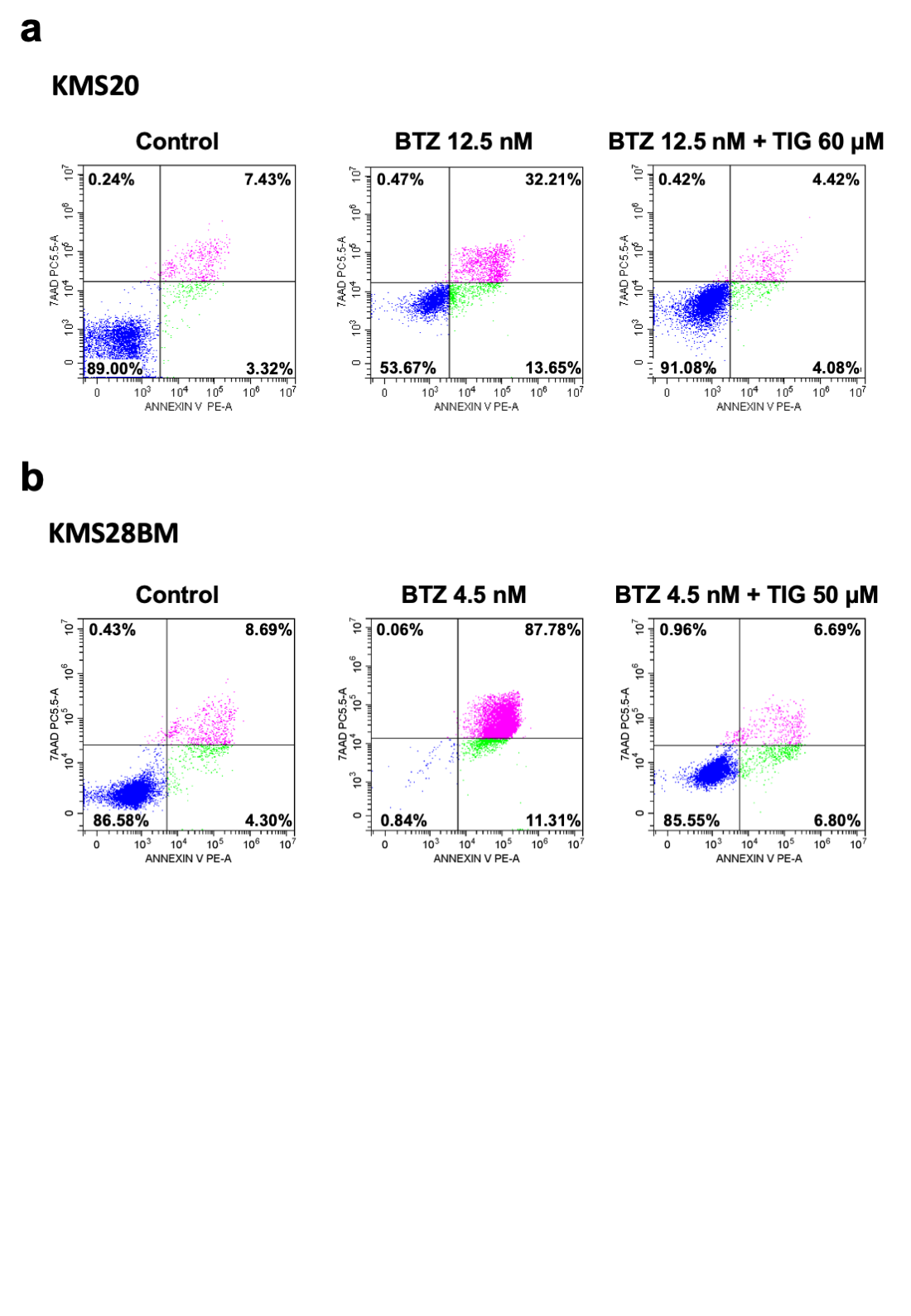

Supplement: Supplementary file 1 [file ijms-25-04887-s001.zip › Figure S1.tiff]

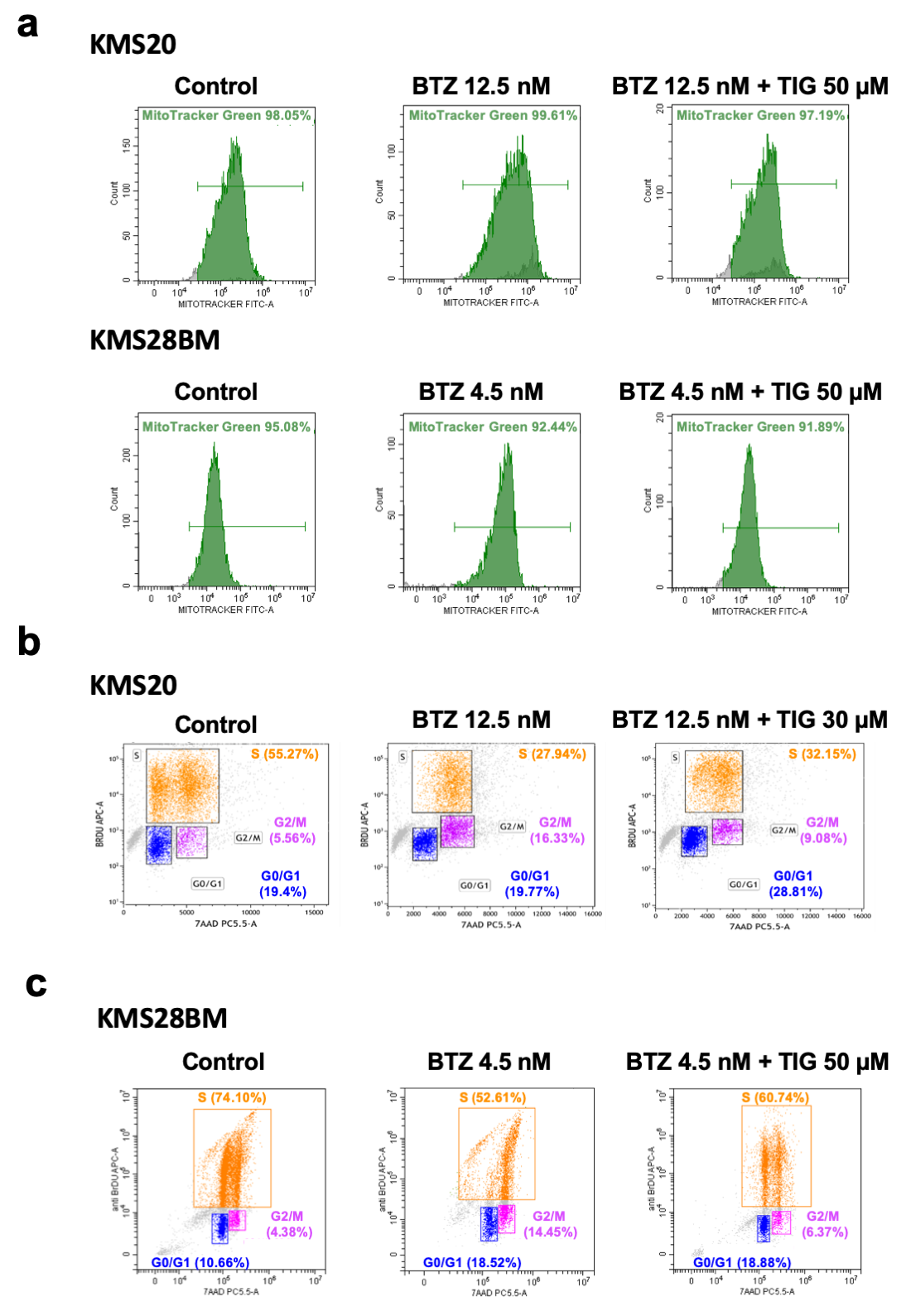

Supplement: Supplementary file 1 [file ijms-25-04887-s001.zip › Figure S2.tiff]

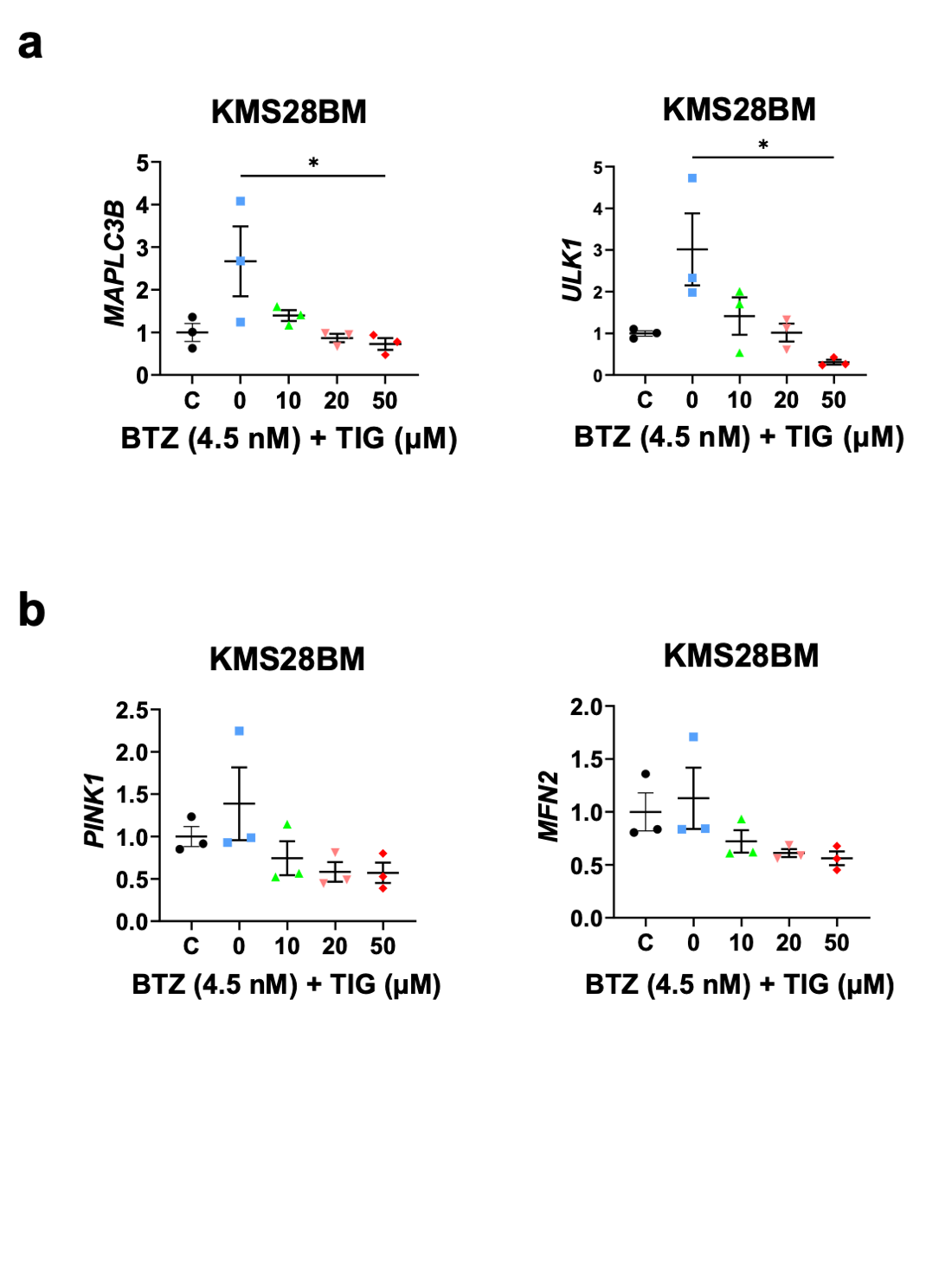

Supplement: Supplementary file 1 [file ijms-25-04887-s001.zip › Figure S3.tiff]

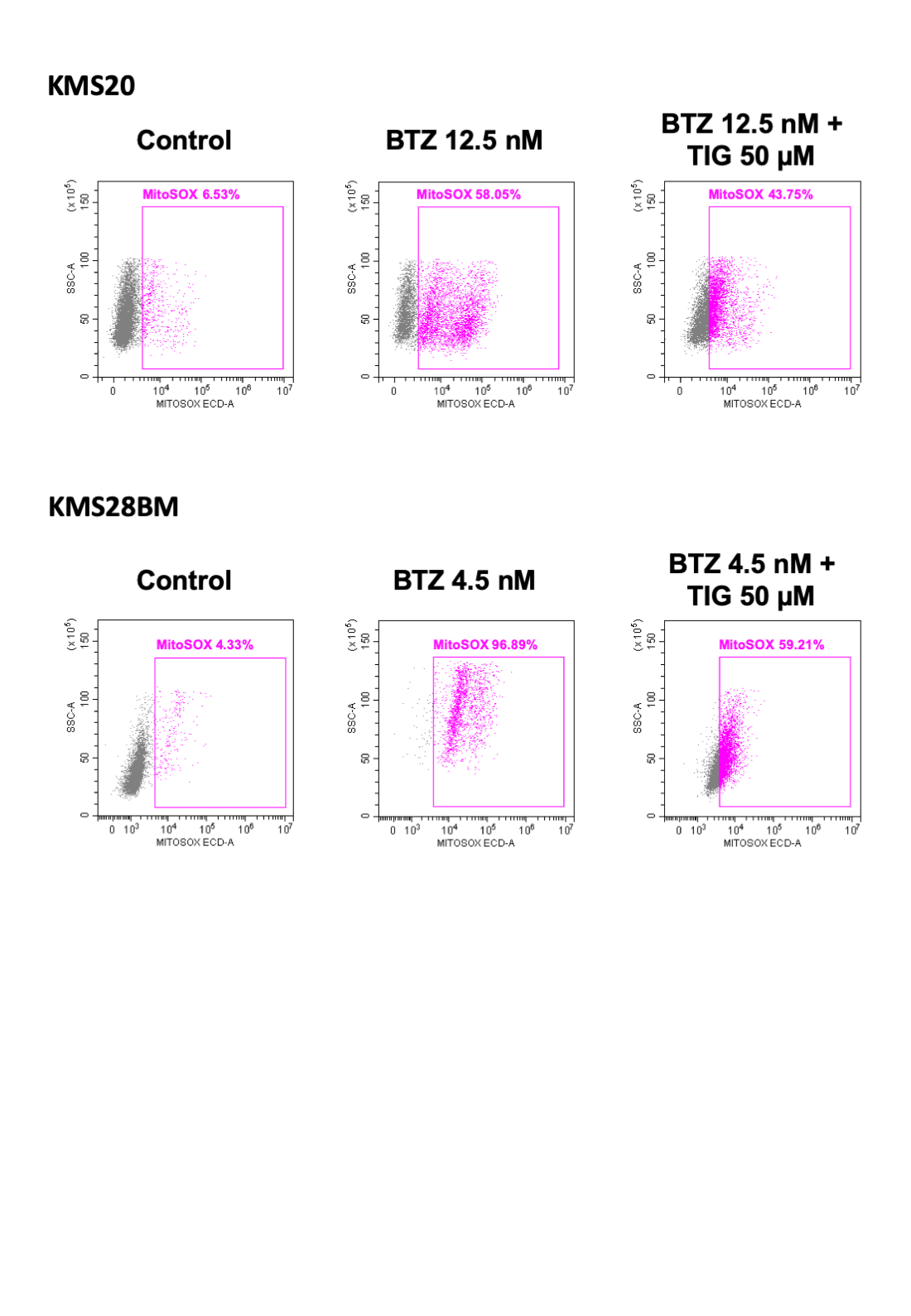

Supplement: Supplementary file 1 [file ijms-25-04887-s001.zip › Figure S4.tiff]

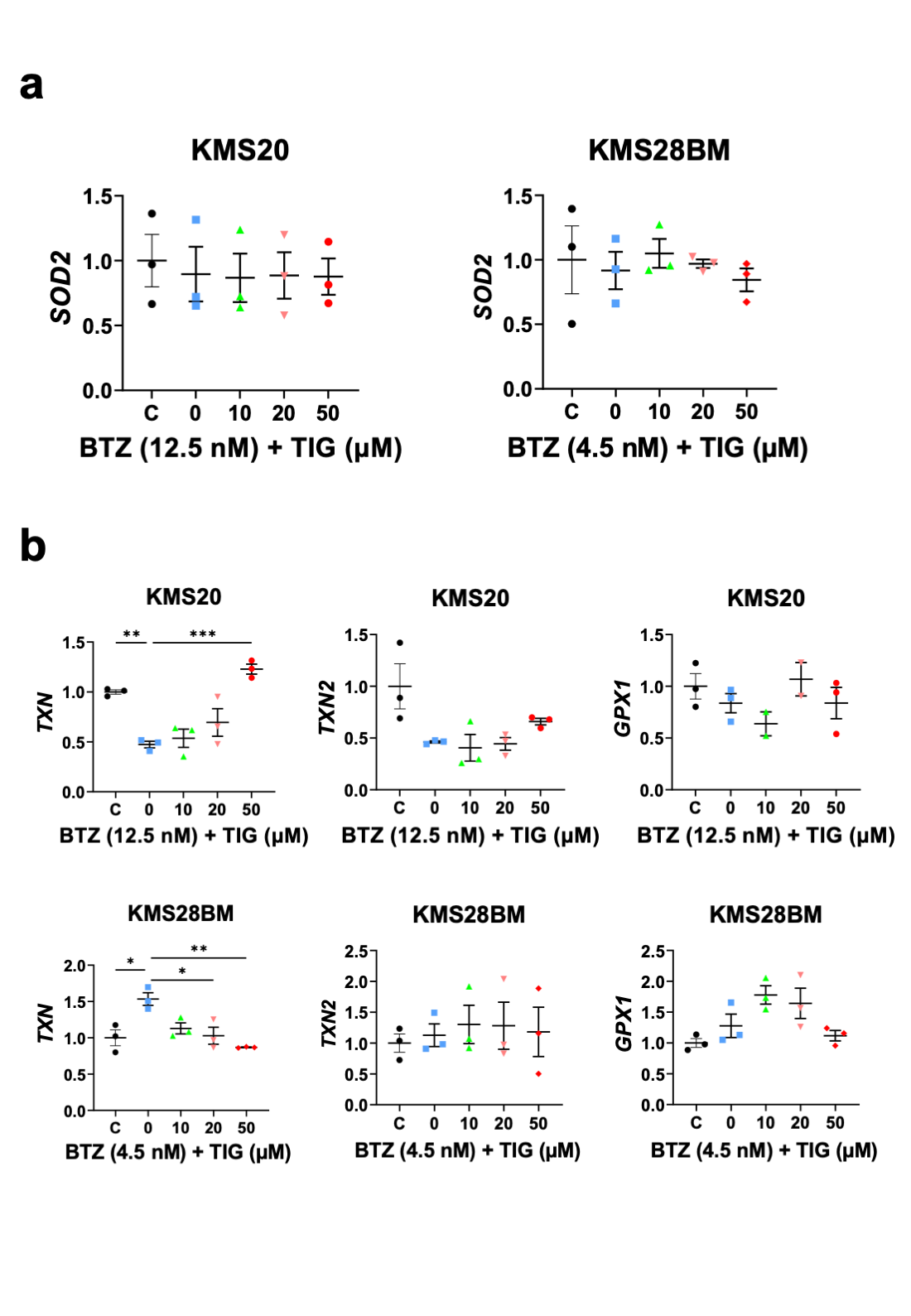

Supplement: Supplementary file 1 [file ijms-25-04887-s001.zip › Figure S5.tiff]

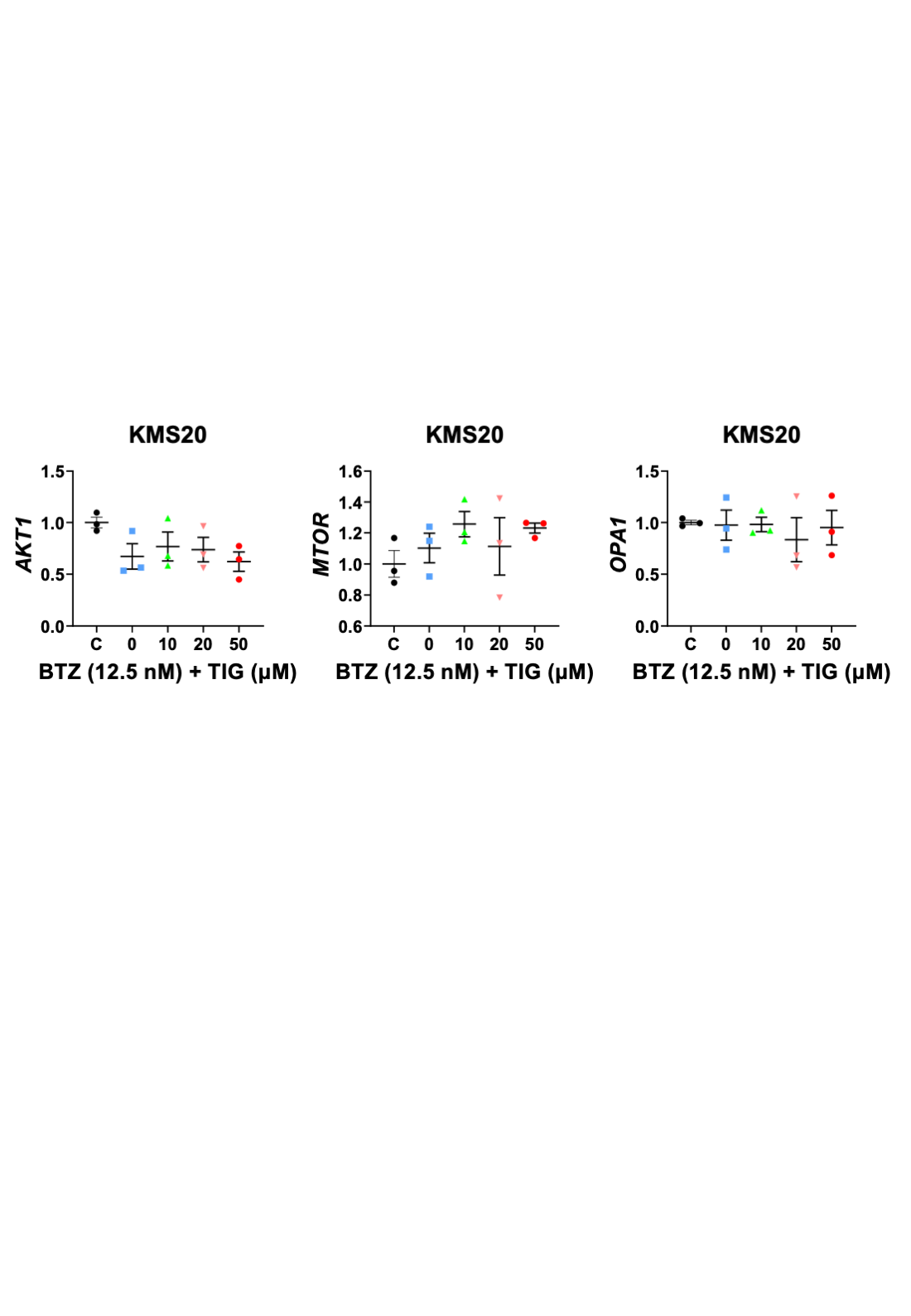

Supplement: Supplementary file 1 [file ijms-25-04887-s001.zip › Figure S6.tiff]

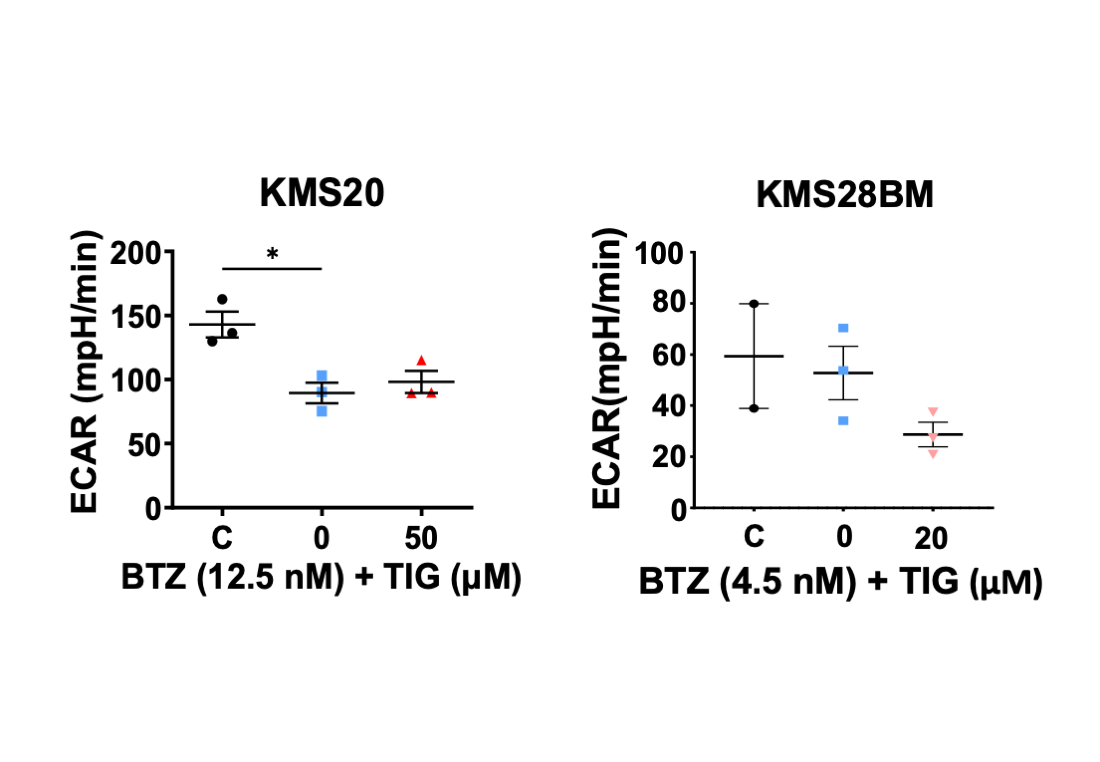

Supplement: Supplementary file 1 [file ijms-25-04887-s001.zip › Figure S7.tiff]

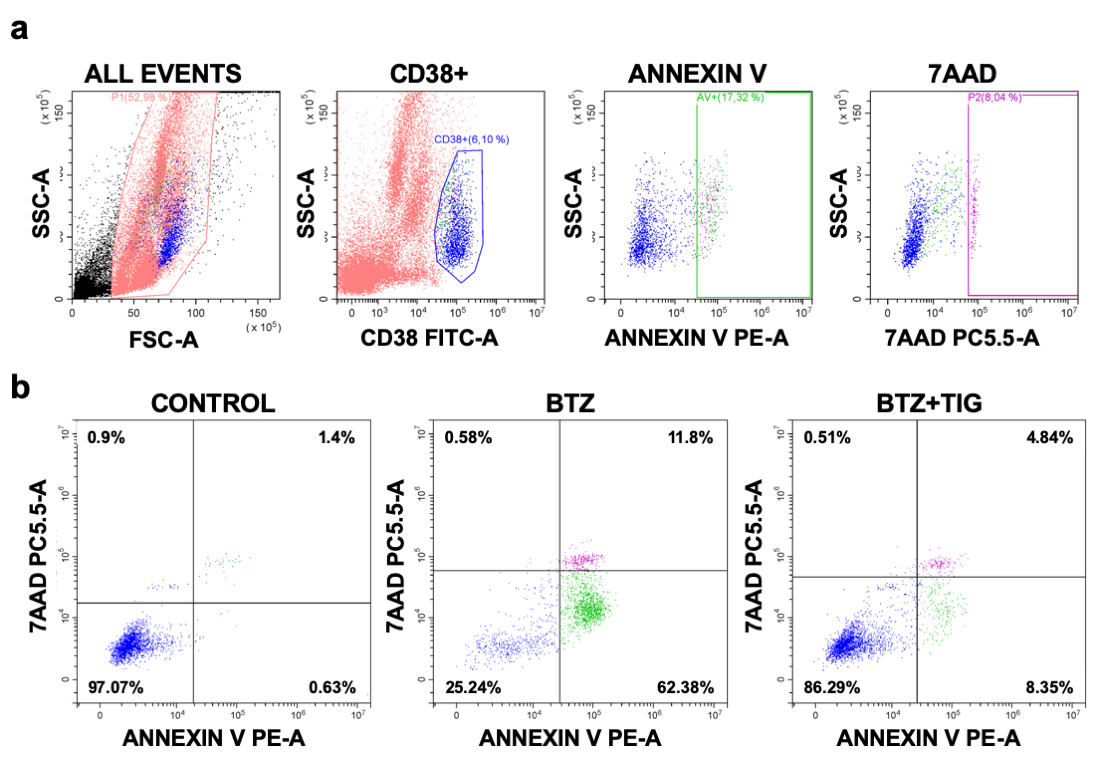

Supplement: Supplementary file 1 [file ijms-25-04887-s001.zip › Figure S8.tiff]

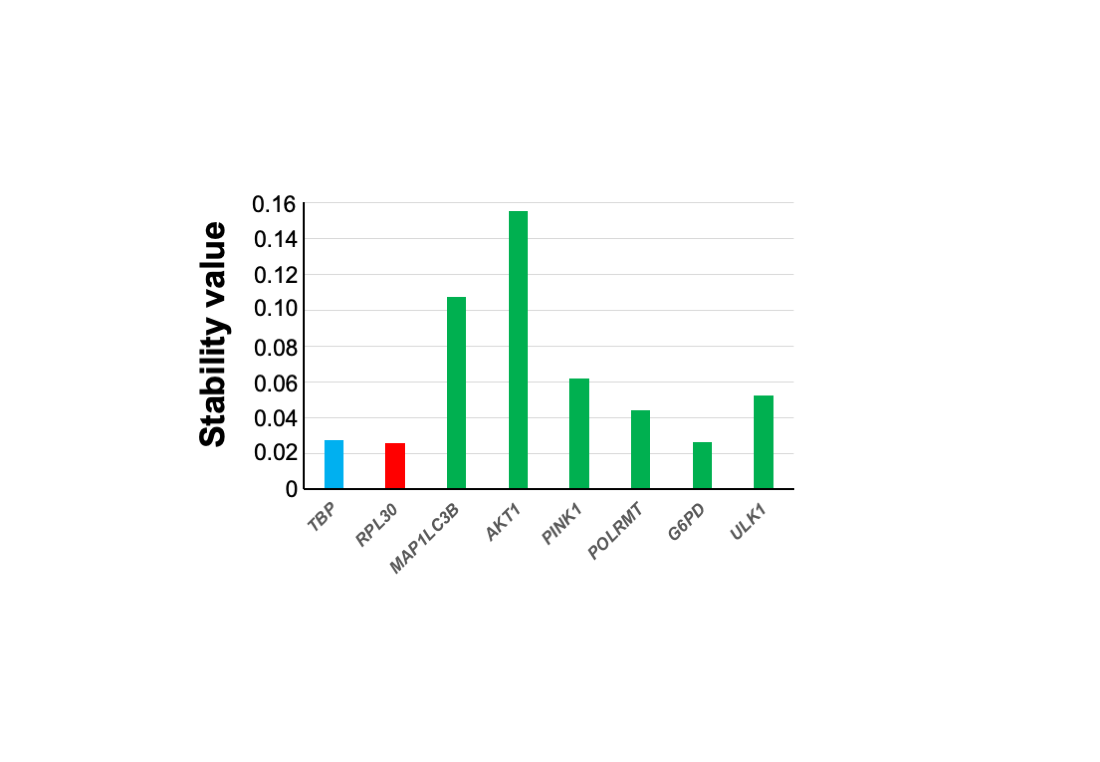

Supplement: Supplementary file 1 [file ijms-25-04887-s001.zip › Figure S9.tiff]
